# Highly recurrent *CBS* epimutations in gastric cancer CpG island methylator phenotypes and inflammation

**DOI:** 10.1186/s13059-021-02375-2

**Published:** 2021-06-01

**Authors:** Nisha Padmanabhan, Huang Kie Kyon, Arnoud Boot, Kevin Lim, Supriya Srivastava, Shuwen Chen, Zhiyuan Wu, Hyung-O K Lee, Vineeth T. Mukundan, Charlene Chan, Yarn Kit Chan, Ong Xuewen, Jason J. Pitt, Zul Fazreen Adam Isa, Manjie Xing, Ming Hui Lee, Angie Lay Keng Tan, Shamaine Ho Wei Ting, Micah A. Luftig, Dennis Kappei, Warren D. Kruger, Jinsong Bian, Ying Swan Ho, Ming Teh, Steve George Rozen, Patrick Tan

**Affiliations:** 1 Programme in Cancer and Stem Cell Biology, Duke-NUS Medical School, 8, College road, Singapore, 169857 Singapore; 2 Centre for Computational Biology, Duke-NUS Medical School, Singapore, 169857 Singapore; 3 Department of Medicine, Yong Loo Lin School of Medicine, National University of Singapore, Singapore, 119228 Singapore; 4 Bioprocessing Technology Institute, A*STAR, 20 Biopolis Way, #06-01 Centros, Singapore, 138668 Singapore; 5 Department of Pharmacology, Yong Loo Lin School of Medicine, National University of Singapore, Singapore, 117600 Singapore; 6 Cancer Biology Program, Fox Chase Cancer Center, Philadelphia, PA USA; 7 Cancer Science Institute of Singapore, National University of Singapore, Singapore, 117599 Singapore; 8 Department of Molecular Genetics and Microbiology, Duke Centre for Virology, Duke University School of Medicine, Durham, NC USA; 9 Department of Biochemistry, Yong Loo Lin School of Medicine, National University of Singapore, Singapore, 117596 Singapore; 10 National University of Singapore (Suzhou) Research Institute, Suzhou, 215123 China; 11 Department of Pathology, National University of Singapore, Singapore, 119228 Singapore; 12 Genome Institute of Singapore, Singapore, 138672 Singapore; 13 SingHealth/Duke-NUS Institute of Precision Medicine, National Heart Centre Singapore, Singapore, 169856 Singapore; 14 Singapore Gastric Cancer Consortium, Singapore, 119074 Singapore; 15 Department of Physiology, National University of Singapore, Singapore, 117593 Singapore

**Keywords:** Gastric cancer, CBS, CIMP, Inflammation

## Abstract

**Background:**

CIMP (CpG island methylator phenotype) is an epigenetic molecular subtype, observed in multiple malignancies and associated with the epigenetic silencing of tumor suppressors. Currently, for most cancers including gastric cancer (GC), mechanisms underlying CIMP remain poorly understood. We sought to discover molecular contributors to CIMP in GC, by performing global DNA methylation, gene expression, and proteomics profiling across 14 gastric cell lines, followed by similar integrative analysis in 50 GC cell lines and 467 primary GCs.

**Results:**

We identify the cystathionine beta-synthase enzyme (CBS) as a highly recurrent target of epigenetic silencing in CIMP GC. Likewise, we show that *CBS* epimutations are significantly associated with CIMP in various other cancers, occurring even in premalignant gastroesophageal conditions and longitudinally linked to clinical persistence. Of note, CRISPR deletion of *CBS* in normal gastric epithelial cells induces widespread DNA methylation changes that overlap with primary GC CIMP patterns. Reflecting its metabolic role as a gatekeeper interlinking the methionine and homocysteine cycles, *CBS* loss in vitro also causes reductions in the anti-inflammatory gasotransmitter hydrogen sulfide (H_2_S), with concomitant increase in NF-κB activity. In a murine genetic model of *CBS* deficiency, preliminary data indicate upregulated immune-mediated transcriptional signatures in the stomach.

**Conclusions:**

Our results implicate *CBS* as a bi-faceted modifier of aberrant DNA methylation and inflammation in GC and highlights H_2_S donors as a potential new therapy for *CBS*-silenced lesions.

**Supplementary Information:**

The online version contains supplementary material available at 10.1186/s13059-021-02375-2.

## Background

The CpG island methylator phenotype (CIMP) is a molecular subtype observed in several malignancies, characterized by aberrant, pervasive, and genome-wide DNA hypermethylation of CpG islands [CGIs]. CGI hypermethylation at gene promoter regions can result in transcriptional gene silencing (“epimutations”), and examples of cancer driver genes inactivated by epimutation include *MLH1* [mutL homolog 1] which can cause microsatellite instability [MSI] and *CDKN2A* [cyclin-dependent kinase inhibitor 2A] resulting in defective cell cycle regulation [1–3]. Recent pan-cancer studies comparing CIMP across multiple tumor types have revealed striking tissue-specific methylation patterns, with minimal conserved hypermethylation [2]. At the pathway level however, analysis of CIMP in various cancers has highlighted common denominators such as the enrichment of PRC2 [polycomb repressive complex 2] targets [4, 5].

Clinically, CIMP in different tumor types has been associated with age, gender, tumor location, histological subtype, survival, and exposure to smoking and infectious pathogens [6]. However, molecular mechanisms driving CIMP remain obscure for most cancer types with very few exceptions. Gain-of-function *IDH1* [isocitrate dehydrogenase (NADP(+)) 1] and loss-of-function *TET* [ten–eleven translocation] mutations have been shown to induce CIMP in one more than one cancer type [2]. Other genes and pathways implicated in CIMP include *SDHB* [succinate dehydrogenase complex iron sulfur subunit B] in paraganglioma [7], *KRAS* [KRAS proto-oncogene, GTPase], and activating *BRAF* [B-Raf proto-oncogene, serine/threonine kinase] mutations in colorectal cancer [8, 9].

Gastric cancer [GC] is a leading cause of global cancer mortality and known to display two distinct CIMP groups—“gastric CIMP” associated with MSI, and “EBV [Epstein-Barr Virus] CIMP” associated with EBV positivity [10]. To date, (epi)genetic drivers of GC CIMP remain largely unknown, although EBV infection is known to induce hypermethylation in vitro [11–13]. Despite their distinct etiologies, gastric CIMP and EBV-CIMP have been reported to share common DNA hypermethylation patterns [11]. However, whether these common hypermethylation patterns are functionally required to contribute and/or sustain features of CIMP, or are mere bystander phenotypes, remains poorly understood. Here, through genome-wide studies of DNA methylation and gene expression patterns in GC cell lines and patient cohorts, we discovered *CBS* [cystathionine beta-synthase] promoter hypermethylation as a highly recurrent epimutation in GC CIMP. We show that loss of *CBS* activity in vitro recapitulates a subset of methylation in GC CIMP tumors, and that besides GC, epimutations at *CBS* are also associated with CIMP in other tumor types, implying a broader role in cancer. Surprisingly, *CBS* loss is also associated with an inflammatory response that may be linked in part to CBS’s ability to regulate H_2_S production.

## Results

### Integrative methylome, transcriptome, and proteome analysis identify highly recurrent *CBS* epimutations in GC CIMP

To identify conserved GC CIMP epimutations, we assembled a panel of 14 gastric cell lines based on previous literature (“Methods”) that comprised 3 gastric/MSI CIMP (NUGC3, NCC59, and IM95), 3 EBV-CIMP (SNU719, YCC10 and NCC24), 6 non-CIMP GC cell lines (SNU16, SNU484, SNU1967, SNU1750, NCC19 and MKN1), and two non-malignant gastric epithelial cell lines (GES1 and HFE145). We verified the CIMP and non-CIMP status of each line based on hierarchical clustering of top variable 10,000 CGI probes derived from Illumina HumanMethylation450 Infinium DNA methylation array data (“Methods,” Additional file 1: Figure S1).

**Fig. 1:**
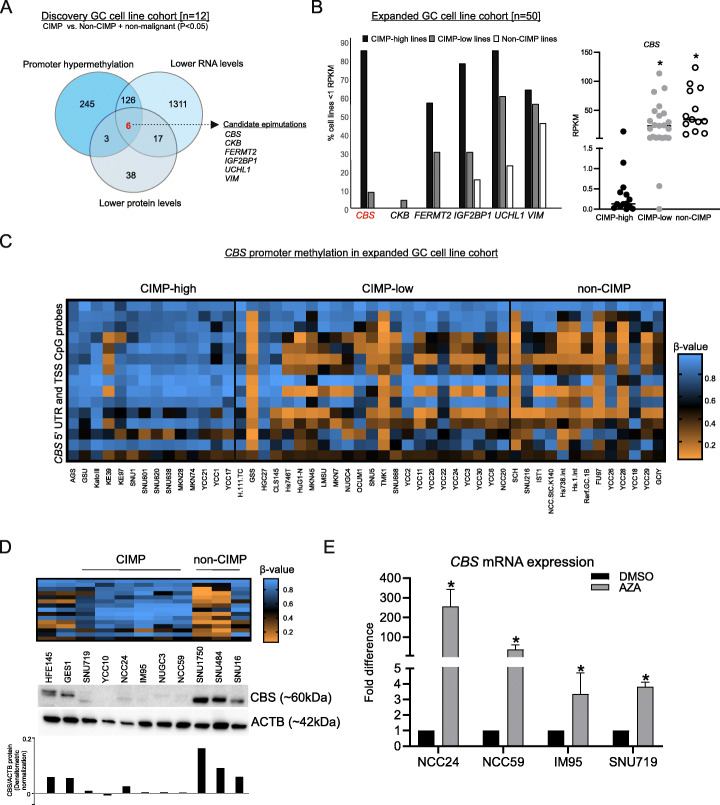
Global analysis identifies *CBS* epimutation as highly conserved in CIMP-positive GC lines. **a** Overlap of genes that are promoter hypermethylated (*P* < 0.05; FDR ≤ 0.1), and downregulated at RNA and protein levels in CIMP vs. non-CIMP GC cell line panel (log_2_ FD ≤ 2, *P* < 0.05; FDR ≤ 0.05) (*n* = 14). **b** Percentage of CIMP-high [*n* = 14], CIMP-low [*n* = 23] and non-CIMP cell lines [*n* = 13] in the expanded GC cell line cohort with RPKM < 1 (gene silencing) for the 6-gene panel listed in **a** [left panel]; RPKM values for *CBS* in the expanded GC cell line cohort (*P < 0.001, Mann-Whitney test; CIMP-high vs. CIMP-low or non-CIMP) [right panel]. **c** Heat map representing promoter methylation *β*-values of *CBS* gene in the expanded GC cell line cohort [*n* = 50] (*P < 0.001, Mann-Whitney test; CIMP-high vs. CIMP-low or non-CIMP). **d** Heat map representing promoter methylation *β*-values of *CBS* gene [upper panel] and CBS protein levels in normal gastric, CIMP, and non-CIMP GC lines using Western blotting and densitometric normalized levels of CBS/ACTB [lower panel]. **e** *CBS* mRNA expression in GC CIMP lines can be re-instated by Azacytidine [*n* = 3] (*P* < 0.05, Student’s *t* test)

We performed global methylation sequencing, RNA sequencing, and proteomic analysis on the 14-cell line panel. Methylomes were interrogated using MeDIP, a technique that can distinguish 5-methyl cytosine from 5-hydroxy methyl cytosine, a demethylation derivative with different transcriptional associations. Combining the gastric CIMP and EBV-CIMP lines, we identified genes exhibiting both aberrant gain of promoter methylation (*P* < 0.05; FDR ≤ 0.1) (Additional file 2) and significant downregulation at the RNA (average log_2_ FD ≤ 2, *P* < 0.05; FDR ≤ 0.05) (Additional file 3) and protein level (average log_2_ FD ≤ 2, *P* < 0.05; FDR ≤ 0.05) (Additional files 4, 5), compared to non-CIMP and non-malignant gastric lines (Fig. 1a). We found a set of 6 candidate epimutations, consisting of *CBS* [cystathionine beta-synthase], *CKB* [creatinine kinase B], *FERMT2* [fermitin family member 2], *IGF2BP1* [insulin like growth factor 2 mRNA binding protein 1], *UCHL1* [ubiquitin C-terminal hydrolase L1], and *VIM* [vimentin] (Fig. 1a).

To establish the most conserved CIMP epimutation among these 6 candidates, we assessed RNA levels of these 6 genes in an expanded cohort of 50 GC cell lines subtyped as “CIMP-high” (*n* = 14), “CIMP-low” (*n* = 23), and “non-CIMP” (*n* = 13) (“Methods,” Additional file 1: Figure S1). A recurrence analysis in these 50 GC cell lines highlighted *CBS* transcriptional loss as the most frequent and specific marker in CIMP-high cell lines (12/14, 85.71%) compared to CIMP-low (2/23, 8.69%) and non-CIMP cell lines (0/13, 0%) (Additional file 6), with significant negative correlations to increased *CBS* promoter methylation (Spearman *r* = − 0.72, *P* < 0.001) (Fig. 1b,c, Additional file 7). Using quantitative PCR and Western blotting, we orthogonally confirmed that *CBS* mRNA and protein are expressed in normal gastric and non-CIMP cell lines but downregulated in CIMP cell lines (Fig. 1d, Additional file 1: Figure S2A, S3; *Refer* Fig. S2B for CBS antibody validation). Treatment of GC CIMP lines with a DNA demethylating agent (Azacytidine) restored *CBS* mRNA expression (FD *=* 3.3–255.8 vs. DMSO, *P* < 0.05), reaffirming the negative relationship between *CBS* epimutation and expression (Fig. 1e) [14]. Since hypermethylated promoter loci in GC are frequently targeted by PRC2 which predominantly catalyzes histone H3 trimethylation on lysine 27 [4, 15], we assessed H3K27me3 status at the *CBS* gene promoter in 2 gastric CIMP cell lines. However, we found no enrichment of this histone mark (Additional file 1: Figure S4), reinforcing a stronger role for DNA methylation in *CBS* silencing, likely in concert with other repressive histone marks (e.g., H3K9me3).

**Fig. 2:**
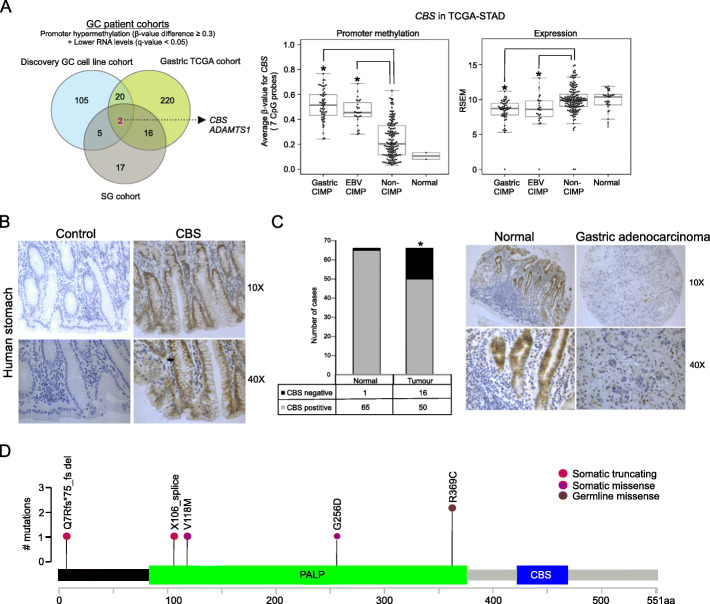
*CBS* epimutations associate with CIMP in primary GCs. **a** Overlap of genes that are promoter hypermethylated and downregulated at the RNA level in the discovery GC cell line panel, TCGA-stomach adenocarcinoma [STAD] and Singapore [SG] cohorts (*β*-value difference ≥ 0.3 and *q*-value < 0.05) [left panel]. Average promoter methylation *β*-values and gene expression of *CBS* gene in STAD according to CIMP subtypes (*P < 0.001, two-tailed Wilcoxon rank sum test, each CIMP group vs. non-CIMP group) [right panel]. **b** Immunohistochemistry of CBS in a normal human stomach with black arrow indicating cytoplasmic staining in epithelial cells. Control sections were not treated with the primary antibody. **c** Summary of CBS staining in 66 cases of matched normal and gastric adenocarcinomas (*P < 0.05, two-tailed Fisher’s exact test) [left panel] and an example of a matched normal vs. tumor case with a negative score [right panel]. **d** Summary of somatic and germline genetic alterations at *CBS* in STAD. PALP, pyridoxal-phosphate dependent enzyme domain; CBS, cystathionine beta-synthase domain; aa, amino acid

Although GC cell lines offer the key advantage of representing a pure population of tumor gastric epithelial cells, we next attempted to validate our findings in primary GCs to eliminate confounders that may have arisen due to in vitro effects. We used a similar approach to analyze methylation array (*β*-value difference ≥ 0.3) and gene expression data (*q*-value < 0.05) in two GC patient cohorts (gastric TCGA [10] and Singapore Cohort [4], *n* = 467 patients). Despite platform differences (“Methods”), this integrative analysis yielded a common signature of 18 genes that included *CBS* (Fig. 2a, Additional file 8). Of the remaining 5 candidate epimutations, *CKB* and *FERMT2* were also significantly associated with CIMP in the gastric TCGA cohort. We verified expression of *CBS* in normal human stomach using quantitative PCR and immunohistochemistry (Fig. 2b, Additional file 1: Figure S2C-D), revealing predominant cytoplasmic staining of CBS in the gastric epithelium, consistent with a previous report in mice [16]. We further tested for CBS protein expression in a tissue microarray comprising 66 cases of matched normal and GCs. A significant proportion of cancer cases exhibited reduced CBS staining in the malignant gastric epithelium (24.2%) compared to matched normal (1.5%) (*P* < 0.05) (Fig. 2c). Unfortunately, associations with CIMP status could not be performed due to lack of methylation data for this GC cohort. These results support *CBS* epimutation as the most recurrent epimutation associated with GC CIMP in both cell lines and primary tumors [14].

We also interrogated the gastric TCGA cohort for somatic and germline mutations in the *CBS* gene. Analysis of 243 GCs identified 6 cases—4 cases with *CBS* somatic mutations all of which were predicted to be truncating or pathogenic (FATHMM score > 0.9), and 2 cases with predicted deleterious germline *CBS* mutations (Fig. 2d, Additional file 9). Mutated cases were heterozygous, with a low prevalence (6/243, 2.46% frequency), co-occurred with gastric CIMP and MSI (Fisher’s exact test, *P* = 0.001), and exhibited promoter hypermethylation (*β*-value range 0.59–0.79), suggesting that genetic mutations are much less frequent than epimutations at the *CBS* locus and may or may not represent passenger events owing to MSI.

### *CBS* epimutations associate with CIMP in multiple cancer types and premalignant intestinal metaplasia

**Fig. 3:**
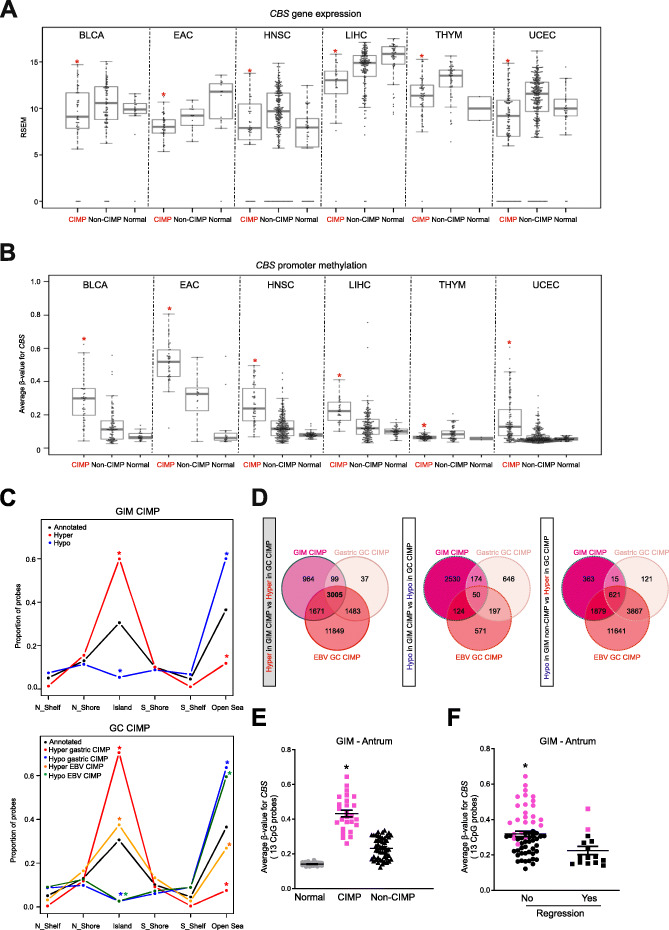
*CBS* epimutation associates with CIMP in multiple tumors and in gastric intestinal metaplasia. **a** Box plot showing *CBS* gene expression levels in CIMP, non-CIMP, and normal categories per cancer type (CIMP vs non-CIMP; *P < 0.05, two-sided Wilcoxon rank sum test). **b** Box plot showing mean promoter methylation *β*-values of *CBS* gene in CIMP, non-CIMP, and normal categories per cancer type (CIMP vs non-CIMP; *P < 0.05, two-sided Wilcoxon rank sum test). **c** Breakdown of GIM CIMP [upper panel] and GC CIMP [lower panel] signatures in CpG contexts island, shore, shelf, and open sea; *P < 0.001 according to binomial test; “Annotated” refers to the distribution of CpG probes on the Infinium methylation array. **d** Three-way Venn overlap of hypermethylated or hypomethylated CpG sites in GIM CIMP and GC CIMP subtypes at CGI level. **e** Average promoter methylation *β*-values of *CBS* gene in normal [chronic gastritis] and antrum GIM lesions based on CIMP (high), and non-CIMP (intermediate) (*P < 0.05 in pair-wise two-tailed Mann-Whitney test with mean *β*-value difference ≥ 0.2 compared to normal group). **f** Average promoter methylation *β*-values of *CBS* gene in GIM cases with or without regression at study-end point; cases in pink belong to CIMP subtype (*P < 0.05 in pair-wise two-tailed Mann-Whitney test)

We then investigated if *CBS* epimutations are associated with CIMP in other cancers. Using TCGA-generated datasets and tumor categorizations of CIMP that has been previously described (“Methods”), we analyzed 22 different cancer types performing *CBS* differential gene expression analysis between most “hypermethylated/CIMP” vs. other methylation categories (Wilcoxon rank sum test, *P* < 0.05, “Methods”). We identified 6 cancers displaying significant downregulation of *CBS* in CIMP vs non-CIMP tumors, including bladder urothelial carcinoma [BLCA] (*P* = 0.02), esophageal adenocarcinoma [EAC] (*P* = 0.01), head and neck squamous cell carcinoma [HNSC] (*P* = 0.006), liver hepatocellular carcinoma [LIHC] (*P* = 3.5 × 10^−5^), thymoma [THYM] (*P* = 3.2 × 10^−6^), and uterine corpus endometrial carcinoma [UCEC] (*P* = 3.6 × 10^−11^) (Fig. 3a). Differential methylation analysis in these 6 cancers further confirmed significant *CBS* promoter hypermethylation in 5/6 of these cancers including BLCA (*P* = 1.1 × 10^−10^), EAC (*P* = 6.1 × 10^−8^), HNSC (*P* = 4.1 × 10^−13^), LIHC (*P* = 2.1 × 10^−9^), and UCEC (*P* = 1.7 × 10^−22^) (Fig. 3b, Additional file 1: Figure S5), with significant inverse correlations to gene expression (Spearman: BLCA *r* = − 0.21, *P* = 0.01; EAC *r* = − 0.46, *P* = 2.1 × 10^−4^; HNSC *r* = − 0.28, *P* = 4.1 × 10^−7^, LIHC *r* = − 0.33, *P* = 9.8 × 10^−8^ ; UCEC *r* = − 0.25, *P* = 1.9 × 10^−7^, Additional file 1: Figure S6) indicating that *CBS* epimutations are associated with CIMP in other tumor types besides GC.

Gastric intestinal metaplasia [GIM] is a premalignant condition of the stomach associated with an estimated annual GC risk of ~ 0.25% [17]. We recently described the existence of CIMP in GIM [15]. Like GC, GIM CIMP is also significantly enriched for hypermethylated CGIs (*P* < 0.001) (Fig. 3c). To assess conserved hypermethylation between GIM and GC CIMP, we compared CGIs (with a minimum of 1 CpG probe overlap) between GIM CIMP, GC CIMP, and non-CIMP groups (*β*-value difference ≥ 0.1, FDR ≤ 0.05; *P* < 1 × 10^−5^, bootstrapping using 100,000 iterations) and identified a “GIM-GC common CIMP signature” of 3005 CGIs (Fig. 3d; Additional file 10). Notably, the GIM-GC common CIMP signature again included aberrant *CBS* promoter hypermethylation (average *β*-value difference = 0.28 between GIM CIMP compared to chronic gastritis in antrum, *P* < 0.05), indicating its early occurrence in the pre-cancerous GC cascade (Fig. 3e). Over a 5-year longitudinal period, GIMs with increased *CBS* promoter methylation were significantly associated with clinical persistence and lack of regression (*P* < 0.05) (Fig. 3f). Similar to GIM and GC, EAC is known to display CIMP and considered to arise from intestinal metaplastic lesions in the esophagus (“Barrett’s esophagus” [BE]). We investigated the methylation status of the *CBS* promoter in BE premalignant lesions using a publicly available dataset [18]. Echoing our observations in GIM, the *CBS* promoter was significantly hypermethylated in BE and EAC but not in control tissues (*P* < 0.05) (Additional file 1: Figure S7).

### Genetic ablation of *CBS* in normal gastric cells results in abnormal DNA methylation

**Fig. 4:**
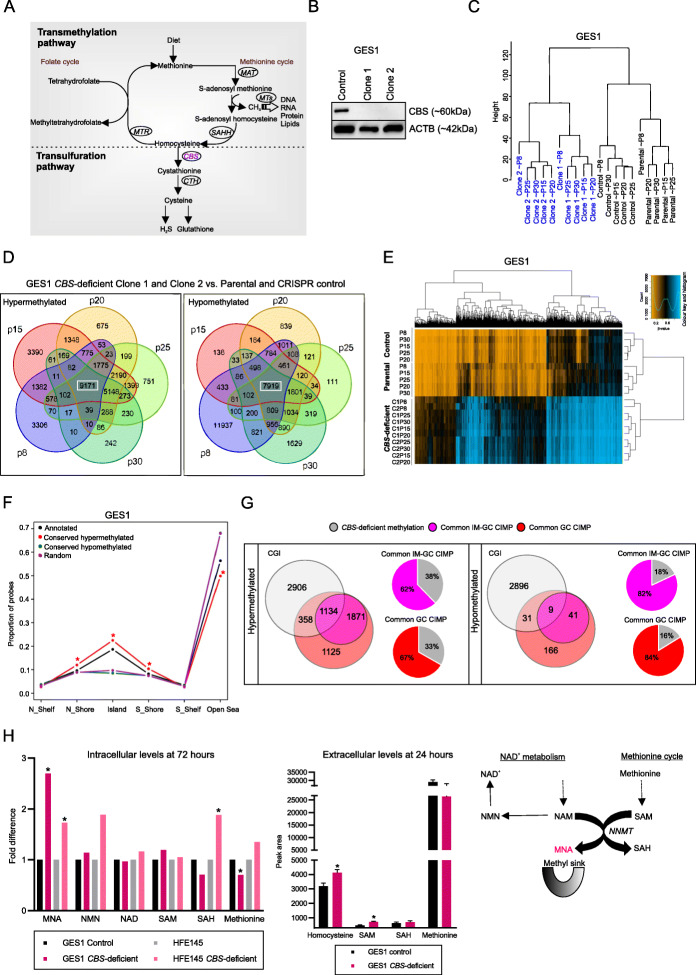
Knocking out CBS alters the DNA methylation landscape in normal gastric epithelial cells. **a** Biochemical position of CBS in the transsulfuration and transmethylation cycles. **b** CBS protein levels in GES1 CRISPR control and *CBS*-deficient CRISPR clones. **c** Cluster dendogram of methylation data indicating branching of GES1 *CBS*-deficient clones from GES1 parental and CRISPR control cells. **d** Venn diagram of differential methylation analysis of GES1 *CBS*-deficient clone 1 and clone 2 compared to parental and CRISPR control cells. **e** Unsupervised clustering of conserved hypermethylated 9171 CpG sites in GES1 *CBS*-deficient clones [clone 1, C1; clone 2, C2] across passages. **f** Breakdown of the GES1 *CBS*-deficient conserved signature in CpG contexts island, shore, shelf, and open sea; *P < 0.001 according to binomial test; “Annotated” refers to the distribution of CpG probes on the Infinium methylation EPIC array, “Random” refers to the sum of all differentially methylated CpG probes in random pairing of samples. **g** Comparison of *CBS*-deficient conserved signatures in GES1 or HFE145 with the “GIM-GC common CIMP” and “GC common CIMP” signatures at CpG islands indicate a larger overlap for hypermethylated (33–38%) events compared to hypomethylation events (16–18%); *P* < 1 × 10^−4^, bootstrapping using 10,000 samples. **h** Graph showing fold difference of intracellular levels of metabolites in *CBS*-deficient cells (*n* = 4–5) compared to controls (*n* = 5) at 72 h, 1-methyl nicotinamide [MNA] (and related metabolites, nicotinamide mononucleotide [NMN], nicotinamide adenine dinucleotide [NAD], S-adenosyl methionine [SAM], S-adenosyl homocysteine [SAH], and methionine, *P < 0.05 (left panel). Graph showing peak area of extracellular levels of homocysteine, SAM, SAH, and methionine in *CBS*-deficient cells (*n* = 5) compared to controls (*n* = 7) at 24 h, *P < 0.05 (middle panel). Biochemical reaction showing conversion of nicotinamide [NAM] to MNA using a methyl group from SAM, catalyzed by nicotinamide N-methyltransferase [NNMT] (right panel). *CBS*, cystathionine beta-synthase; *CTH*, cystathionine gamma-lyase; *MTR*, 5-methyltetrahydrofolate-homocysteine methyltransferase; *MAT*, Methionine adenosyltransferase; *MT*s, Methyl transferases

CBS encodes an enzyme occupying a unique biochemical position in the methionine and homocysteine metabolic pathways (Fig. 4a). Homocysteine is converted into methionine and subsequently S-adenosyl methionine [SAM], the universal methyl donor for DNA, RNA, histones, and lipids. Because CBS can break down homocysteine into cystathionine, we reasoned that CBS loss may lead to homocysteine accumulation and disrupt normal methyl group homeostasis, and potentially implicate *CBS* loss-of-function epimutations as more than a marker of GC CIMP. This is also supported by the fact that *CBS-*deficient mice display significant plasma homocysteinemia (where excess cellular homocysteine is exported), and tissue-specific metabolic defects in the methionine pathway [19]. To investigate this possibility, we employed CRISPR genome editing to generate *CBS* loss-of-function models in a non-malignant gastric epithelial cell line (GES1), using two different guide RNAs (Fig. 4b; Additional file 1: Figure S3).

To study DNA methylation in GES1 *CBS*-deficient clones, we used Infinium methylation EPIC arrays to assay GES1 parental, CRISPR control with a non-targeting sequence, *CBS*-deficient clone 1, and *CBS*-deficient clone 2 across 5 different passage numbers (~P8 to P30, culture time ≤ 3 months). Hierarchical clustering of GES1 parental, CRISPR control, and *CBS*-deficient clones indicated a clear divergence of the latter, suggesting that CBS loss induces genome-wide methylome changes (Fig. 4c). To minimize clonal effects, we performed differential methylation analysis of GES1 *CBS*-deficient clones (*n* = 10) compared to parental and CRISPR control cells (*n* = 10), such that a minimum *β*-value difference ≥ 0.2 was present in both clones compared to all controls at each passage number. This analysis indicated a conserved signature of 17,090 CpG sites (9171 hypermethylated and 7919 hypomethylated CpG sites; Fig. 4d,e, Additional file 11), which was absent in random pairings of samples (Additional file 1: Figure S8). Importantly, this conserved hypermethylation exhibited features of in vivo CIMP, including enrichment for CGIs and depletion of open sea regions (*P* < 0.001), and enrichment of PRC2 targets (*P* < 0.001) (Fig. 4f, Additional file 1: Figure S9F). Analysis of hypermethylated CpG sites with gene expression indicated passenger and non-passenger methylation events, with a distinct subset of genes downregulated (FD ≤ 0.6) in both GES1 *CBS*-deficient clones in the latter category (6.8%, 76/1155 genes) (Additional file 12). These included cancer epigenetic targets previously implicated in tumor suppressive activity such as *ANXA6* [annexin A6] [20], *VANGL2* [VANGL planar cell polarity protein 2] [21], *BIN1* [bridging integrator 1] [22], and *CREB3L1* [cAMP responsive element binding protein 3 like 1] [23].

To rule out cell-line-specific effects, we generated *CBS*-deficient clones in another non-malignant gastric epithelial cell line (HFE145), performing a similar methylation analysis at two different passage numbers (P5 and P23) (Additional file 1: Figures S9A, S3). Differential methylation analysis of HFE145 *CBS*-deficient clones compared to parental and CRISPR control cells yielded a conserved signature of 6886 CpG sites across both passages (Additional file 1: Figure S9B-C, Additional file 13). Although the magnitude of change in HFE145 *CBS*-deficient clones was milder than that of GES1 *CBS*-deficient clones, the conserved hypermethylation was still enriched for CGIs and depleted for open sea regions (*P* < 0.001), and enriched for PRC2 targets (*P* < 0.001) (Additional file 1: Figure S9D-F). Analysis of hypermethylated CpG sites with gene expression again indicated passenger and non-passenger methylation events, with 6.1% (29/468 genes) of genes downregulated (FD ≤ 0.6) in both HFE145 *CBS*-deficient clones (Additional file 14).

To evaluate the concordance of DNA hypermethylation targets derived in vitro due to *CBS* deficiency and in vivo CIMP profiles, we compared the signatures observed in *CBS*-deficient clones (GES1 and/or HFE145) with the previously identified “GIM-GC common CIMP signature.” We observed a significant ~ 38% overlap at CGIs (*P* < 1 × 10^−4^, bootstrapping using 10,000 samples, Fig. 4g, Additional file 15) that included candidate tumor suppressor *ROBO1* [roundabout guidance receptor 1] [24, 25] that is known to be promoter hypermethylated and inactivated in multiple cancers.

Since we observed a DNA methylation phenotype in *CBS*-deficient cells, we also asked if CBS deficiency caused metabolic changes in gastric epithelial cells, particularly in metabolites related to the methylation pathway. We profiled ~ 244–327 compounds (polar and lipid) in cellular extracts from GES1 and HFE145 cell line models at 72 h and performed differential analyses of *CBS*-deficient clones compared to parental and CRISPR control cells (Additional file 16, “Methods”). We identified 8 metabolites altered in *CBS*-deficient clones of both cell lines (4 increased, 4 decreased). Interestingly, these included increased levels of “1-methyl nictotinamide” [MNA] (FD 1.7–2.6; *P* < 0.05), a metabolite linked to the SAM cycle and considered a “methyl sink” (Fig. 4h, Additional file 1: Figure S10A), where excess methyl groups from SAM are transferred to nicotinamide (Fig. 4h, left and right panels) [26–28]. The mRNA expression of nicotinamide N-methyltransferase enzyme that catalyzes this reaction remained unchanged in *CBS*-deficient clones (Additional file 1: Figure S10B), implying that increased MNA levels reflect disturbances in methyl group homeostasis rather than enzyme expression levels. In addition, we also observed multiple triglycerides and phospholipids exhibiting significant changes, consistent with a prior study in *CBS*-deficient mice that reported that CBS loss alters lipid metabolism [29] (Additional file 1: Figure S10C). Of note, intracellular homocysteine could not be detected in this global analysis, likely due to the sensitivity of platform to detect low levels at 72 h [30]. Since excess homocysteine is usually exported out of the cell, we then measured homocysteine, SAM, S-adenosyl homocysteine [SAH], and methionine in culture media from the GES1 cell line model at 24 h using targeted assays (“Methods”). We found significant increases in homocysteine (FD 1.28; *P* < 0.05) and SAM (FD 1.50; *P* < 0.05), but not SAH and methionine in *CBS*-deficient cells (Fig. 4h, middle panel), further suggestive of an imbalance in the methyl group cycle. Taken collectively, we find that loss of *CBS* in two independent normal gastric epithelial cell lines was associated with an abnormal methylation landscape that overlapped with in vivo CIMP patterns and an altered metabolic profile.

### *CBS* loss is also associated with an inflammatory state

**Fig. 5:**
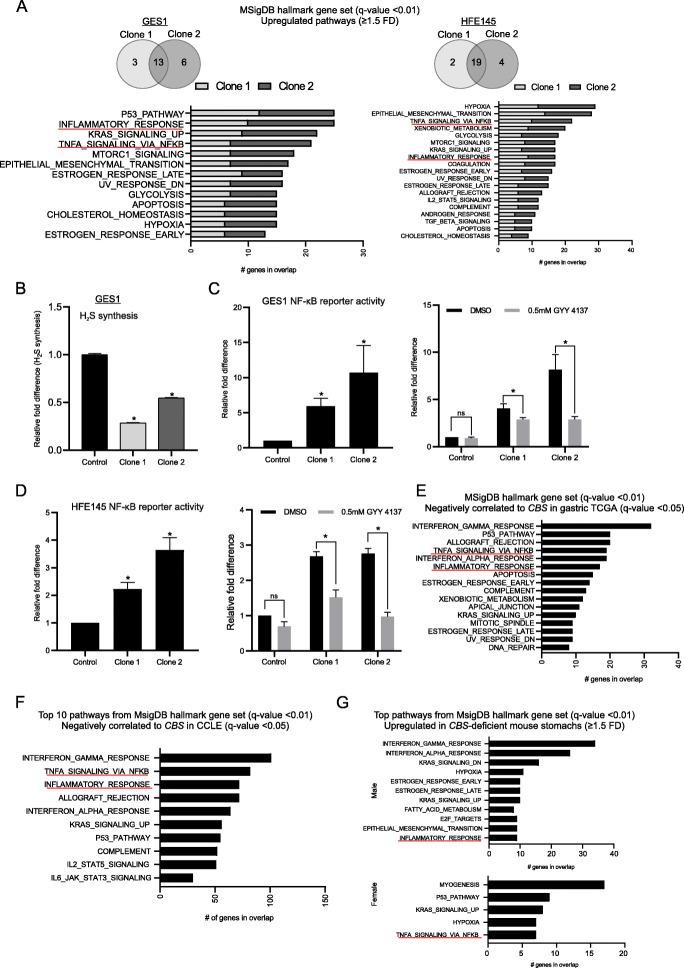
Loss of CBS is also associated with an inflammatory response. **a** Overlap of upregulated MSigDB hallmark pathways in GES1 and HFE145 *CBS*-deficient clones and the list of shared pathways; “k” is the number of genes in the intersection of the MSigDB hallmark query set (*q*-value < 0.01). **b** Relative H_2_S levels in GES1 *CBS*-deficient clone 1 [28%] and clone 2 [54%] compared to CRISPR control cells [*n* = 3] (*P < 0.05). **c** NF-κB luminescence reporter assay in GES1 *CBS*-deficient cells, and in **d** HFE145 *CBS*-deficient cells, at 48 h [*n* = 3] and in 0.5 mM GYY 4137-treated cells at 24 h [1 = 3]. Control represents data pooled from parental and CRISPR control cells (*P < 0.05). **e** Upregulated MSigDB hallmark pathways (*q*-value < 0.01) in genes negatively correlated to *CBS* gene expression in gastric TCGA (*q*-value < 0.05) and in **f** CCLE (*q*-value < 0.05). **g** Upregulated MSigDB hallmark pathways (*q*-value < 0.01) in mouse *CBS*-deficient stomachs (FD (≥ 1.5))

Unexpectedly, besides DNA methylation alterations, RNA sequencing analysis of both GES1 and HFE145 *CBS*-deficient clones compared to parental and CRISPR control cells revealed an upregulation of inflammation-related pathways, such as “*Inflammatory response*” and “*TNFA signalling via NF-κB*” (*q*-value < 0.01) (Fig. 5a, Additional files 17 and 18). To reaffirm this finding, we measured NF-κB activity (pro-inflammatory complex) in GES1 and HFE145 *CBS*-deficient clones using a reporter assay and observed significantly higher activity in these cells (5.9–10.7 FD in GES1; 2.2–3.6 FD in HFE145, compared to parental and control CRISPR cells, *P* < 0.05) (Fig. 5c, left panel; Fig. 5d, left panel).

To understand this phenomenon in greater detail, we revisited the CBS metabolic network and noted that besides being linked to transmethylation, CBS also catalyzes the first step of the transsulfuration pathway, leading to the generation of hydrogen sulfide [H_2_S] and glutathione [31]. Consistent with the functional loss of CBS, both GES1 *CBS*-deficient clones exhibited significantly lower H_2_S levels (0.28–0.54 FD compared to CRISPR control cells, *P* < 0.05) (Fig. 5b). H_2_S is a gasotransmitter, and the CBS-H_2_S axis has been implicated in multiple physiological processes including inflammation, vasodilation, and cell metabolism, in part through H_2_S’s function of “sulfhydration”—a post-translational modification on proteins [32–34]. We thus reasoned that the induction of inflammatory pathways in *CBS*-deficient cells could in part be caused by reductions in H_2_S levels [35]. Treatment with a H_2_S slow-release donor GYY 4137 at 0.5 mM for 24 h significantly ameliorated NF-κB activity in *CBS*-deficient cells (1.4–2.8 FD in GES1; 0.3–0.5 FD in HFE145, compared to DMSO control, *P* < 0.05) (Fig. 5c, right panel; Fig. 5d, right panel), supporting the idea that loss of H_2_S in *CBS*-deficient cells contributes to a portion of the inflammatory response.

To validate these in vitro findings in primary GCs and other data sets, we identified genes that were negatively correlated in expression to *CBS* in the gastric TCGA cohort (*q*-value < 0.05). We found that these genes significantly overlapped with inflammation-related pathways in the MSigDB hallmark gene sets that included “*Inflammatory response*” and “*TNFA signalling via NF-κB*” (*q*-value < 0.01) (Fig. 5e, Additional file 19). Likewise, in BLCA, ESCA, HNSC, and LIHC, genes that were negatively correlated in expression to *CBS* (*q*-value < 0.05) significantly overlapped with “*Inflammatory response*” pathways (*q*-value < 0.01) (Additional file 20). A similar gene expression analysis from CCLE [*n* = 1739 cell lines [36] again confirmed that genes negatively correlated in expression to *CBS* (*q*-value < 0.05) overlapped with inflammation-related pathways including “*Inflammatory response*” and “*TNFA signalling via NF-κB*” (*q*-value < 0.01) (Fig. 5f, Additional file 21).

Finally, we explored the consequences of *CBS* deficiency in vivo using *Tg-hCBS Cbs*^−/−^ knockout mice (“Methods”) [37]. Hematoxylin and eosin staining of *Cbs*^−/−^ stomachs (*n* = 11; aged 2.1 to 11.1 months) vs. *Cbs*^+/+^ stomachs (*n* = 6; aged 2.8–8.1 months) showed no overt histological defects or neoplasia at this age (data not shown). We performed RNA sequencing in age-matched *Cbs*^−/−^ (*n* = 2; 1 male, 1 female) and *Cbs*^+/+^ (*n* = 2; 1 male, 1 female) stomachs for differential gene expression analysis (FD ≥ 1.5 and FD ≤ 0.6) (Additional file 22). As expected, *CBS* mRNA levels were lower in *Cbs*^−/−^ stomachs (FD = 0.08–0.14 compared to *Cbs*^+/+^ stomachs, Additional file 22). Despite low sample numbers, pathway analysis using PANTHER (FDR < 0.05) identified “circadian regulation of gene expression” as the top upregulated pathway, consistent with a recent study reporting a distinct role for CBS in circadian function [38]. More pertinent to this study, several immune-related processes were also significantly upregulated including “complement activation” and “defense response to bacterium,” indicative of an inflammatory state in *CBS*-deficient stomachs (Additional file 23) and concurring with our earlier observations. In addition, pathway analysis using MSigDB hallmark gene sets also indicated “*Inflammatory response*” and “*TNFA signalling via NF-κB*” as significantly upregulated (*q*-value < 0.01) (Fig. 5g; Additional file 24). A similar PANTHER (FDR < 0.05) pathway analysis of downregulated genes in *Cbs*^−/−^ stomachs did not yield significant hits.

## Discussion

Using a multi-platform approach comprising methylation, RNA, and protein datasets to identify “epimutations” in GC, we determined *CBS* as a recurrent and conserved epigenetic target in CIMP tumors, associated with both MSI and EBV GC. In vitro loss of CBS in normal human gastric epithelial cells is associated with both aberrant DNA methylation and an inflammatory response, indicating a novel bi-faceted function to this epimutation. Whether these in vitro phenotypes are observed in mouse models, and/or exacerbate with prolonged periods of time, following a “methylation accumulation model” needs to be ascertained, given that primary lesions also take years to develop. Nevertheless, the dual phenotypes are pertinent given a long-standing link between CIMP lesions and inflammation, with chronic inflammation considered a universal accelerator of DNA hypermethylation [39–41]. Notably, pan-cancer analysis indicated that these associations were observed in other tumor types beyond GC, implying a broader role for *CBS* epimutation in shaping tumor biology. The tissue-independent mechanism leading to epigenetic targeting of *CBS* is still unknown. It may be triggered either by a fundamental cellular insult, dysregulation of factors impacting *CBS* expression including non-coding RNAs [42], or perhaps arises as an adaptation to disturbances in biochemically connected pathways such as methionine levels [43, 44]. It is relevant to point out that prior cancer studies have reported both loss and overexpression of the *CBS* gene, relating to tumor suppressive and oncogenic properties respectively, with the latter associated with an abnormal bioenergetic phenotype [45]. We also observe high levels of CBS expression in a proportion of GC cell lines that tend to be non-CIMP (Fig. 1b), suggesting that *CBS* gene dosage is a key factor to maintain a normal metabolic state, and its expression distinctly varies with tumor subtype.

We demonstrated that *CBS* deficiency in normal gastric cell lines resulted in increased homocysteine, SAM, and MNA (“methyl sink”), findings also seen in other models where methylation patterns are abnormal and indicative of altered methyl group homeostasis [26, 27]. While our study has not identified the exact mechanism by which *CBS* deficiency results in abnormal DNA methylation, we speculate that the phenomenon may be complex, involving metabolic perturbations in the transmethylation cycle (e.g., other classes of methyl group acceptors such as histones, RNA, lipids), and perhaps also the transsulfuration pathway (e.g., H_2_S, glutathione, inflammatory changes) since several of these components have been previously identified as modifiers of DNA methylation, through direct and indirect mechanisms [46–51]. Further functional studies that mimic the metabolic states associated with *CBS* deficiency (e.g., homocysteine exposure) and culture manipulations are required to identify the precise metabolic sequelae leading to altered methylation. For example, it will be useful to assess metabolic changes in *CBS*-deficient cells grown in media with lower methionine levels, since normal media is considered to contain supraphysiological levels of methionine and could thus blunt the severity of phenotypes assessed [26]. Interestingly, recent studies in colorectal cancer implicated the process of ageing as a key contributor to CIMP, where spontaneous ageing of normal colon organoids led to a CIMP-like state with enhanced susceptibility to transformation by *BRAF*^V600E^ mutations, in turn enhancing the CIMP phenotype [8]. A pan-cancer analysis also found that a majority of CIMP tumors have increased DNA methylation drift, also associated with ageing [52]. Of note, *CBS* downregulation was observed in gene expression signatures associated with methylomic drift by two independent studies in esophageal and colorectal cancers [53, 54]. *CBS* has also been directly connected to ageing in fruit flies, wherein *CBS* overexpression increased lifespan [55]. Whether age-related processes play a causative or consequential role to *CBS* epimutation also needs to be examined. A limitation of our study is that the current experimental design may not accurately model fine-scale methylation changes across time, as this will involve a larger set of biological replicates/passage with extended culture times.

Our findings in *CBS*-deficient gastric cells that lower H_2_S levels are associated with increased NF-kB activity, draws from multiple studies in other cell line and animal model systems that have investigated this mechanistically [35, 56–58]. These previous studies have shown the anti-inflammatory function of *CBS* loss and H_2_S to be multifaceted, involving the following: blocking NF-kB p65 activation, regulating cellular redox status, DNA damage, stabilization of hypoxia-inducible factor (HIF)-1α, regulating immune cell migration, mitochondrial electron transport, and apoptosis. At the molecular level, H_2_S’s role in “sulfhydration,” has been linked to some of these biological effects [59]. Future work in the *CBS*-deficient in vitro model will systematically investigate the role of these molecular mechanisms in inducing inflammatory responses. Preliminary investigations in *CBS*-deficient mouse stomachs indicated upregulation of several immune-related processes. In line with these findings, previous studies have reported an inflammatory phenotype in bone, liver, and kidney of *CBS*-deficient mice [34, 60]. Of significance, inflammatory markers of osteoporosis in *CBS-*deficient mice could be dampened using H_2_S donor treatment, synchronous with our data that showed a significant rescue of NF-κB activation in *CBS*-deficient human gastric cells. A similar protective effect of H_2_S donors have also been reported in lipopolysaccharide-induced inflammation, rodent models of gastric ulcer, and BE [33, 61, 62], and in preventing viral infections [63]. Based on these collective findings, we think that an in-depth characterization of *CBS-*deficient and GIM mouse models to malignancy risk, inflammation, and H_2_S releasing therapeutics [64] is further warranted.

Finally, a common CIMP signature between GC and GIM CIMPs is reminiscent of findings from other premalignant settings (e.g., BE and EAC) [18] and supports the “DNA methylation accumulation model.” It also prompts assessment of the existence of CIMP and *CBS* epimutations in preceding lesions to address the chronology of molecular events. Even so, *CBS* promoter hypermethylation was significantly associated with the clinical persistence of GIM lesions. We hypothesize that loss of CBS in early gastric lesions could be one of the mechanisms by which abnormal DNA methylation and inflammatory state is induced, which over several years and compounded by other risk factors, ultimately leads to accrual of pro-tumorigenic DNA methylation changes and unresolved chronic inflammation (Additional file 1: Figure S11). It is also possible that *CBS* epimutation likely precedes EBV onset since GIMs are not clonally infected, and perhaps may even contribute to lowered cellular defense to viral infection. It will be valuable to test these ideas using clinical samples and appropriate mouse models.

## Conclusion

In summary, our study discovered a novel association between *CBS* epimutations and CIMP subtype in GC, with in vitro models of *CBS* deficiency resulting in abnormal DNA methylation and inflammatory response. These findings provide new insights on the CIMP epigenetic class and re-emphasize epimutations as key players in gastric tumor biology.

## Materials and methods

### Cell lines and primary tissue

Cell lines were purchased from Japan Health Science Research Resource Bank (IM95, NUGC3, and MKN1), ATCC (SNU16, AGS) and Korean Cell Line Bank (NCC59, NCC24, SNU1967, SNU719, SNU484, NCC19, and SNU1750). YCC10 and YCC11 (Yonsei Cancer Centre in Seoul, South Korea), GES1 (Dr. Alfred Cheng, Chinese University of Hong Kong), and HFE145 (Dr. Hassan Ashktorab, Howard University) were kind gifts. Cell lines were authenticated using Short Tandem Repeat profiling using ANSI/ATCC ASN-0002-2011 guidelines and tested Mycoplasma negative according to the MycoAlert Mycoplasma Detection Kit (Lonza). Normal human stomach antrum and fundus tissue slides were purchased from Novus Biologicals (NBP2-30203, NBP2-30204). Human tissue microarray slides containing matched normal and gastric cancer cases, and human control tissue sections (liver and muscle) were provided and processed by SingHealth Advanced Molecular Pathology Laboratory, Singapore.

### GC cell line panel and subtyping

EBV and MSI positive and negative cell lines (*n* = 12) for the GC discovery cell line panel were selected based on prior literature [65–69]. For the DNA methylation subtyping of GC cell lines (*n* = 62), raw intensity data from the Infinium HumanMethylation450 beadchip array were analyzed in R using the minfi package to obtain beta values (ratio of methylation). Samples were normalized using the functional normalization function. CpG sites mapping to sex chromosomes or SNP regions were excluded. Similar to the criteria used in the gastric TCGA study, variations (quantified by standard deviations) of the methylation levels at CpG sites within CpG islands across all samples were calculated, and the top 10,000 sites (Additional file 1: Figure S1) were used for an unsupervised hierarchical clustering using Ward’s linkage method and Euclidean distance metrics which identified 4 major branches. We categorized these 4 branches into 3 major methylation groups, which we termed “CIMP-high” (branch 1), “CIMP-low” (branches 3 and 4), and “non-CIMP” (branch 2). Infinium HumanMethylation450 beadchip array data and mutational status of select cancer genes for the GC cell lines was accessed through the Singapore Gastric Cancer Consortium (http://www.sgcc.sg/research/unique-resources).

### MeDIP-sequencing and analysis

DNA was sonicated using COVARIS S2 and peak fragment distribution between 100 and 500 bp was verified on an Agilent Bioanalyzer (Agilent Technologies) using the DNA1000 chip. Fragmented DNA was end-repaired, dA-tailed, and adapter ligated using NEBNext® DNA Library Prep Master Mix Set for Illumina (E6040). Samples were then spiked with control DNAs that were unmethylated, methylated, and hydroxymethylated (Diagenode C02040010) as a quality control measure. For each sample, input DNA that was not exposed to the primary antibody was included. Adapter-ligated DNA was subjected to immunoprecipitation with a primary monoclonal antibody against 5-methyl cytosine (Diagenode C15200081) using a previously published protocol [70]. Real-time PCR using primers against the spiked DNA controls were performed to verify successful and specific enrichment of methylated DNA (data not shown). Immunoprecipitated samples were amplified using Phusion® High-Fidelity DNA Polymerase (M0530) and NEBNext® Multiplex Oligos for Illumina® (E7335) for 10 cycles. Amplified libraries were run on the Agilent Bioanalyzer using the High sensitivity DNA kit prior to Illumina sequencing using a single-end 100 base pair configuration. MeDIP short reads were aligned to the h19 reference genome and duplicates were removed using SAM tools. Average library size of the 14 MeDIP libraries were ~ 80 million reads. Peaks were called using MACS2 using input control. Differential peak analysis between cell lines was performed using DiffBind (FDR ≤ 0.1) of CIMP vs. non-CIMP and non-malignant cell lines which generated a list of 595 unique genes with gain of promoter methylation (+/− 2 kb TSS) and annotated using GenCODE.

### RNA sequencing and analysis

Total RNA quality check was done using the RNA 6000 Lab Chip Kit on the Agilent Bioanalyzer (Agilent Technologies, Palo Alto, CA). Libraries were prepared using Illumina Tru-Seq Stranded Total RNA with Ribo-Zero Gold kit protocol, according to the manufacturer’s instructions (Illumina, San Diego, CA, USA). Library fragment size was determined using the DNA 1000 Kit on the Agilent Bioanalyzer (Agilent Technologies). Libraries were quantified by qPCR using the KAPA Library Quantification Kit (KAPA Biosystems). Libraries were pooled in equimolar and cluster generation was performed on the Illumina cBOT system (Illumina). Sequencing (150 bp pair-end) was performed on the Illumina HiSeq 3000 system at the Duke-NUS Genome Biology Facility, according to the manufacturer’s protocol. The paired end reads were aligned to the human reference genome (GRCh37) using Hisat2. Gene expression profile (FPKM or RPKM) for each sample was determined.

### Proteomics and analysis

Each cell line was grown and extracted in quadruplicates using RIPA buffer (Sigma) according to the manufacturer’s instructions. In total, 200 μg of protein was used for MS sample preparation. Samples were boiled at 95 °C prior to separation on a 12% NuPAGE Bis-Tris precast gel (Thermo Fisher Scientific) for 15 min at 170 V in 1× MOPS buffer. The gel was fixed using the Colloidal Blue Staining Kit (Thermo Fisher Scientific), and each lane was divided into 2 equal fractions. For in-gel digestion, samples were destained in destaining buffer (25 mM ammonium bicarbonate; 50% ethanol), reduced in 10 mM DTT for 1 h at 56 °C followed by alkylation with 55 mM iodoacetamide (Sigma) for 45 min in the dark. Tryptic digest was performed in 50 mM ammonium bicarbonate buffer with 2 μg trypsin (Promega) at 37 °C overnight. Peptides were desalted on StageTips and analyzed by nanoflow liquid chromatography on an EASY-nLC 1200 system coupled to a Q Exactive HF mass spectrometer (Thermo Fisher Scientific). Peptides were separated on a C18-reversed phase column (25 cm long, 75 μm inner diameter) packed in-house with ReproSil-Pur C18-AQ 1.9 μm resin (Dr Maisch). The column was mounted on an Easy Flex Nano Source and temperature controlled by a column oven (Sonation) at 40 °C. A 215-min gradient from 2 to 40% acetonitrile in 0.5% formic acid at a flow of 225 nl/min was used. Spray voltage was set to 2.4 kV. The Q Exactive HF was operated with a TOP20 MS/MS spectra acquisition method per MS full scan. MS scans were conducted with 60,000 at a maximum injection time of 20 ms and MS/MS scans with 15,000 resolution at a maximum injection time of 50 ms. The raw files were processed with MaxQuant version 1.5.2.8 using the LFQ quantification option [71] on unique+razor peptides with at least 2 ratio counts. Carbamidomethylation was set as fixed modification while methionine oxidation and protein N-acetylation were considered as variable modifications. Search results were filtered with a false discovery rate of 0.01. Known contaminants, proteins groups only identified by site, and reverse hits of the MaxQuant results were removed. LFQ intensities can be found in Supplementary data file (GC Proteomics). Differential analysis (log base_2_ fold change < 2, adjusted *p* value < 0.05) revealed 64 downregulated proteins in GC CIMP [*n* = 6] vs non-CIMP and non-malignant cell lines [*n* = 8] (Additional files 4, 5).

### Gene expression and pathway analysis

Differential gene expression of GC CIMP [*n* = 6] vs non-CIMP and non-malignant cell lines [*n* = 8] was performed using Deseq2 (log base_2_ fold change ≤ 2, adjusted *p* value < 0.05). For the pan-cancer analysis, 22 cancers (TCGA abbreviations: LIHC, HNSC, ESCA, THYM, BLCA, UCEC, ACC, LGG, BRCA, CESC, CHOL, COAD-READ, GBM, KIRP, LUAD, MESO, PAAD, PRAD, SARC, SKCM, TGCT, and UVM) were analyzed for differential gene expression analysis of *CBS* in most “hypermethylated/CIMP” vs. other methylation categories (Wilcoxon rank sum test, *P* < 0.05), as defined by TCGA (The Cancer Genome Atlas) or by Karpinski et al (for UCEC) [52] via MEXPRESS [72]. For UCEC, the TCGA-CIMP sample labels were not publicly available, and therefore, we used CIMP sample labels from Karpinski et al (a pan-cancer study of CIMP). For the GES1 *CBS*-deficient cells (P15) and HFE145 *CBS*-deficient cells (P23), a log base_2_ fold change was generated for each clone compared to parental and CRISPR control cells and further filtered with a minimum difference of 1 FPKM (fragments per kilobase of exon model per million reads mapped). Only genes with a linear fold difference (upregulated [1.5 fold change]) and downregulated [0.6 fold change]) were used for the gene set overlap analysis. For TCGA and CCLE (Cancer Cell Line Encyclopedia) data, genes positively and negatively correlated with *CBS* expression were obtained from cbioportal and filtered using *q*-values < 0.05. MSigDB overlap was computed with hallmark gene sets (v6.2) with *q*-values < 0.01. For the mouse samples, a log base_2_ fold change was generated for each gender in the *Tg-hCBS Cbs*^*-/-*^ category compared to *Cbs*^*+/+*^and further filtered with a minimum difference of 0.5 FPKM. Differential analysis revealed 413 upregulated (1.5 fold change) and 527 downregulated genes (0.6 fold change) common to both genders, which was used for the PANTHER Go-Slim Biological process analysis (FDR < 0.05). For the MSigDB analysis of mouse samples, a log base_2_ fold change was generated for each gender in the *Tg-hCBS Cbs*^*-/-*^ category compared to *Cbs*^*+/+*^and further filtered with a minimum difference of 1 FPKM. Differential analysis and conversion of mouse to human gene identifiers by the MSigDB tool (v7.2) indicated the following. For upregulated genes (1.5 fold change), 412/1032 converted in female and 555/1249 converted in male whereas for downregulated genes (0.6 fold change), 962/1768 converted in female and 558/1191 converted in male. Overlap with hallmark gene sets (v7.2) with *q*-values < 0.01 was performed with the converted IDs.

### Identification of candidate epimutations in CIMP group

For the discovery GC cell line panel, we overlapped promoter hypermethylated genes (*n* = 595), with those downregulated at RNA (*n* = 1464 genes) and protein levels (*n* = 64) in CIMP vs. non-CIMP groups using omic platforms as described in the relevant section. For the recurrence analysis of the candidate epimutations in the expanded GC cell line cohort, RPKM, and methylation values were accessed through the Singapore Gastric Cancer Consortium (http://www.sgcc.sg/research/unique-resources). For the independent analysis of primary GCs, promoter associated hypermethylated genes in CIMP (as defined by TCGA and clustering of Singapore cohort methylation data [4]) vs. non-CIMP group (*β*-value difference ≥ 0.3) were overlapped with downregulated genes in CIMP vs non-CIMP groups (*q*-value < 0.05, fold difference ≤ 0.6, median RPKM value in non-CIMP is ≥ 1). We used a stringent *β*-value difference of ≥ 0.3 since we were concomitantly assessing the effect of hypermethylation on gene expression. TCGA methylation and gene expression data were derived from Infinium HumanMethylation450 beadchip and RNA sequencing platforms respectively. SG cohort methylation and gene expression data were derived from Infinium HumanMethylation27 beadchip and Affymetrix Human Genome U133 Genechips platforms respectively. Except for *FERMT2* in SG cohort, CpG probes pertaining to the other candidate epimutations were represented in both cohorts.

### RNA extraction, reverse transcription, and quantitative PCR

Total RNA was extracted using the Qiagen RNAeasy mini kit according to the manufacturer’s instructions. RNA was converted to cDNA using Improm-II^TM^ Reverse Transcriptase (Promega). Quantitative PCR was performed in triplicate using Quantifast SYBR Green PCR kit (Qiagen) on an Applied Biosystems HT7900 Real Time PCR System for 40 cycles using the following primers: *CBS* Exon 10/11 F—5′ TGCGGAACTACATGACCAAG 3′ , *CBS* Exon 10/11 R—5′ TGAGGTCCTCCTCCTTCAGA 3′, *CBS* Exon 16/17 F—5′ GCAGATCCAGTACCACAGCA 3′, *CBS* Exon 16/17 R—5′ CGAAGGAGAAGTGGGCAAG 3′, *ACTB* F—5′ TCCCTGGAGAAGAGCTACG 3′, *ACTB* R—5′ GTAGTTTCGTGGATGCCACA 3′. Delta Ct was calculated as a difference in Ct number between experimental and reference gene.

### Western blotting

Protein lysates were prepared using ice-cold RIPA buffer (Thermo Fisher Scientific, 89900) containing protease inhibitor cocktail (Nacalai Tesque). Protein concentration was determined using Pierce BCA protein assay (Thermo Scientific, 23225). *Laemmli sample buffer* with reducing agent was added to the lysates and boiled at 100 °C for 5 min. Samples were loaded in each well of a 4–15% Mini-Protean TGX gel (Bio-Rad) and transferred to the PVDF membrane using a semi-dry blotting system (Bio-Rad). Membranes were probed with monoclonal antibodies against CBS (1 in 500 dilution, Abnova H00000875-M01) or ACTB (1 in 2000 dilution, Sigma A5316) in 5% milk-PBST [phosphate-buffered saline with Tween] at 4 °C overnight. Secondary anti-mouse antibody (LNA931, Amersham) was used at a dilution of 1 in 2000 for 1 h at room temperature. Membranes were developed using Amersham ECL Prime Western Blotting Detection Reagent and imaged using Chemidoc Imaging system (Bio-Rad). Bands were quantified using Image Lab software (Bio-Rad).

### *CBS* genetic data

Somatic mutation and copy number data for the stomach adenocarcinoma TCGA 2014 dataset was obtained from cbioportal. FATHMM scores for the single-nucleotide *CBS* somatic variants were derived from COSMIC database. Germline exomes for the stomach TCGA cohort were aligned and variant called as previously described [73]. Briefly, variants with an ExAC MAF < 0.05 were predicted to be deleterious if they were HIGH impact (via Variant Effect Predictor) or MODERATE impact with a CADD score > 25. An additional pathogenicity score for rs117687681 (R369C) was computed using CScape (0.89). *CBS* promoter methylation values for the 6 mutated cases were calculated as an average of 2 TSS probes, cg22633722 and cg09622447.

### H3K27me3 chromatin immunoprecipitation [ChIP]

H3K27me3 ChIP was performed by Zymo Research (ab6002, Abcam). After recovery of ChIP and input DNA, whole-genome amplification was performed using the WGA4 kit (Sigma-Aldrich) and BpmI-WGA primers. Amplified DNAs were purified using PCR purification columns (QIAGEN) and digested with BpmI (New England Biolabs) to remove WGA adapters. Thirty nanograms of amplified DNA was used for each sequencing library preparation (New England Biolabs). Libraries were sequenced on Hiseq2500 (Illumina) to an average depth of 20–30 million reads per library. Sequence reads were mapped against the human reference genome (hg19) using Burrows-Wheeler Aligner (BWA-MEM, version 0.7.10). Reads from histone ChIP-seq were trimmed at the first and last 10 bp prior to alignment. Reads with low mapping quality (MAQP< 10) and PCR duplications were removed using Samtools. Histone ChIP-enriched peaks were detected using CCAT. Positive controls for H3K27me3 enrichment were selected from a prior study [74].

### Transfection experiments

SNU1750 cells were transfected with 3 individual siRNAs for *CBS* (Integrated DNA Technologies, hs.Ri.CBS.13.1/13.2/13.3]) or negative control (Integrated DNA Technologies, 51-01-14-04) at 5 nM using Lipofectamine RNAiMAX (Thermo Fisher Scientific, 13778150). RNA and protein were harvested at 48 h. For the NF-κB reporter assay [75], GES1 and HFE145 *CBS*-deficient cell lines were transfected with 500 ng of 6X-NF-κB-Luciferase reporter or promoter-less pGL3-Basic vector using Lipofectamine 2000 (Thermo Fisher Scientific). Luciferase activity (Promega, E1500) was measured at 48 h post transfection for basal measurements and at 24 h post transfection for the GYY 4137 experiment. Fold change in luciferase activity was normalized to the viability of the cells using the CellTiter-Glo® Luminescent cell Viability Assay for all samples.

### Cell line treatment with drug

CIMP cell lines were treated with 1uM Azacytidine [Sigma-Aldrich, A3656] or DMSO for 72 h prior to RNA harvest. GES1 and HFE145 *CBS*-deficient cell lines were treated with 0.5 mM of H_2_S donor GYY 4137 (Cayman Chemical, 13345) or DMSO for 24 h.

### Immunohistochemistry

CBS monoclonal antibody (Abnova H00000875-M01) was initially tested for immunohistochemistry application in different buffer and antigen retrieval conditions using human liver sections as a positive control (data not shown), after which the following automated method was selected on the Leica Bond III machine. In brief, antigen retrieval was performed with Bond Epitope Solution 2, with primary antibody dilution of 1 in 400 and incubation time of 20 min. Leica Bond Refine kit was used for detection. Imaging was performed on a Nikon Eclipse Ti. The gastric tissue microarray slides were analyzed by a pathologist and cases were scored “positive” if cytoplasmic staining was found in > 5% of gastric epithelial cells and “negative” if staining was entirely absent or present in < 5% of gastric epithelial cells.

### Generation of *CBS* CRISPR clones

GES1 and HFE145 cells were transiently transfected with CRISPR Cas single format vector with a backbone of U6-gRNA/CMV-Cas9-GFP (Sigma-Aldrich) and containing guide RNAs targeting exon 3 of *CBS* gene (HS0000002142 [clone1], HS0000002144 [clone 2], Sigma-Aldrich) or control vector (CRISPR universal negative control 1, Sigma-Aldrich) using Lipofectamine 2000. After 48 h, ~ 960 GFP positive single cells from each transfection were sorted by the Duke-NUS Flow core facility. After ~ 3 weeks, at least ten clones from each vector were cultured and screened and knockouts verified by loss of CBS protein using Western blotting. For each cell line model, 2 *CBS*-deficient clones, 1 CRISPR control, and parental cells were utilized for all downstream experiments.

### Metabolic analysis of *CBS* CRISPR clones

GES1 parental, CRISPR control cells, and *CBS*-deficient clone 1 and clone 2 were cultured and harvested together at P16–P19. HFE145 parental, CRISPR control cells, and *CBS*-deficient clone 1 and clone 2 were cultured and harvested together at P23–P24. Each cell line was harvested at least in duplicate. Cells were collected and quenched using 150 mM cold sodium chloride (Merck, Germany) solution. Following centrifugation at 3400*g*, the cell supernatant was removed. The cell pellets obtained were then processed using a two-phase liquid–liquid extraction protocol based on the modified method of Bligh and Dyer. Briefly, polar and lipid metabolites were extracted by sequential addition of methanol (Optima grade, Fisher Scientific, USA), 3.8 mM tricine (Merck) solution and chloroform (gradient grade, Merck) (1:0.5:1 v/v/v, total 2 mL) to the sample. The mixture was vortexed for 1 min following each addition of solvent. The mixture was then centrifuged at 4 °C, 14,000*g* for 20 min. This resulted in separation of the sample into two fractions—the top methanol-tricine solution layer contained the polar metabolites while the bottom chloroform layer contained the lipid species. The top polar layer was collected and the remaining mixture was re-extracted using a mixture of methanol and tricine solution (9:10 v/v), followed by centrifugation at 4 °C, 14,000*g* for another 10 min. The resulting polar layer was combined together with the first polar extract and the bottom chloroform layer containing the lipid metabolites was collected separately. Both layers were stored at – 80 °C before LC-MS analysis. Each polar extract was analyzed in triplicate using an ultra-performance liquid chromatography system (UPLC, Acquity, Waters, USA) in tandem with a mass spectrometer (Q Exactive, Thermo Scientific, USA). A C18 UPLC column (Acquity UPLC HSS T3 column, 2.1 × 100 mm, 1.8 μm, Waters) was used for separation and the mobile phase comprised of two solvents. “A” being water with 0.1% formic acid (VWR, USA) and “B” being methanol with 0.1% formic acid. The UPLC program is as follows: the column was first equilibrated for 0.5 min at 0.1% B. The gradient was then increased from 0.1% B to 50% B over 8 min before being held at 98% B for 3 min. The column was washed for a further 3 min with 98% acetonitrile (Merck) with 0.1% formic acid and finally equilibrated with 0.1% B for 1.5 min. The solvent flow rate was set at 0.4 mL/min; a column temperature of 30 °C was used. The eluent from the UPLC system was directed into the MS. High-resolution mass spectrometry was then performed in both positive and negative electrospray ionization (ESI) modes, with a mass range of 70 to 1050 m/z and a resolution of 70,000. Sheath and auxiliary gas flow was set at 30.0 and 20.0 (arbitrary units) respectively, with a capillary temperature of 400 °C. The spray voltages were 1.25 kV for positive and 1.5 kV negative mode ionization. Mass calibration was performed using standard calibration solution (Thermo Scientific) prior to injection of the samples. A quality control (QC) sample comprising of equal aliquots of each sample was run at regular intervals during the batch LC-MS runs. Lipid extracts were analyzed in triplicate using an ultra-performance liquid chromatography system (UPLC, Acquity, Waters) in tandem with a mass spectrometer (Xevo G2 QToF, Waters). A C18 UPLC column (Acquity UPLC CSH column, 1.0 × 50 mm, 1.7 μm, Waters) was used for separation and the mobile phase comprised of two solvents: “A” comprising of acetonitrile, methanol, and water (2:2:1) with 0.1% acetic acid (Merck) and 0.025% ammonia solution (VWR, USA), and “B” comprising of isopropanol (Fisher Scientific) with 0.1% acetic acid and 0.025% ammonia solution. The UPLC program is as follows: the column was first equilibrated for 1 min at 1% B with a flow rate of 0.1 mL/min. The gradient was then increased from 1% B to 82.5% B over 9 min before B was further increased to 99% for a 5 min wash at a flow rate of 0.15 mL/min. The column was re-equilibrated for 2.2 min at 1% B. Column temperature was maintained at 45 °C and eluent from the LC system was directed into the MS. High-resolution mass spectrometry was then performed in both positive and negative ESI modes with a mass range of 100 to 1800 m/z and a resolution of ≥ 10,000. Cone and desolvation gas flows was set at 40.0 and 600.0 (L/h) respectively, with a desolvation temperature of 600 °C. The ESI capillary voltages were 2.0 kV for positive mode ionization, and 1.0 kV for negative mode ionization. Mass calibration was performed using sodium formate prior to injection of the samples. Similarly, a quality control (QC) sample comprising of equal aliquots of each sample was run at regular intervals during the batch LC-MS runs. The raw LC-MS data obtained from both polar and lipid extracts was processed using a XCMS-based peak finding algorithm. The QC samples were first used to adjust for instrumental drift and subsequently the experimental data was normalized based on the total ion intensity. Metabolite identities were confirmed based on mass spectral comparison with available metabolite standards. Both univariate (Student’s *t* test with Welch correction) and multivariate (Partial Least Squares Discriminant Analysis, PLS-DA) (SIMCA version 13.0.3, Umetrics, Sartorius, Sweden) approaches were used to identify statistically significant differences in metabolite levels between parental and CRISPR control cells vs. *CBS*-deficient clone 1 and clone 2 (univariate—*p* value < 0.05; multivariate—variable importance for projection, VIP > 1).

For the spent media sample preparation, GES1 parental, CRISPR control cells, and *CBS*-deficient clone 1 were cultured and harvested together at P20. RPMI media containing no cells was used as a control. Twenty microliters of 0.5 M dithiothreitol (DTT) (Bio-Rad, CA, USA) was added to 400 μL of medium [30, 76]. The mixture was vortexed for 1 min and incubated at 37 °C for 15 min. After incubation, the mixture was centrifuged at 4 °C, 12,000*g* for 5 min. The treated medium was transferred into a vial and used immediately for homocysteine detection using LC-MS/MS analysis. For the detection of SAM, SAH, and methionine, the spent medium was analyzed separately without any treatment. The treated medium was analyzed in 4 replicates using an ultra-performance liquid chromatography system (UPLC, Acquity, Waters Corp) in tandem with a triple-quadrupole mass spectrometer (Xevo TQ-S, Waters Corp) operating in ESI-positive mode. The source temperature and desolvation temperature were set at 150 °C and 500 °C, respectively. The cone gas flow was 150 l h^–1^ and the desolvation gas flow was 700 l h^–1^. The capillary voltage was 2.90 kV. Compound-dependent MS parameters and multiple-reaction monitoring transitions for the analytes are shown in the table below. A C18 UPLC column (Acquity UPLC BEH column, 2.1 × 100 mm, 1.7 μm, Waters) was used for chromatographic separation and the mobile phase comprised of two solvents: “A” comprising of water with 0.1% formic acid (VWR, USA) and “B” comprising of methanol (Fisher Scientific, USA) with 0.1% formic acid. The UPLC program is as follows: the column was first equilibrated for 0.5 min at 0.1% B with a flow rate of 0.4 mL/min. The gradient was then increased from 0.1% B to 10% B over 2.5 min before B was further increased to 50% for 3 min at a flow rate of 0.3 mL/min. The column was re-equilibrated at 0.4 mL/min for 1.5 min at 1% B. Column temperature was maintained at 30 °C and eluent from the LC system was directed into the ESI source of the mass spectrometer. The injection volume was 4 μl. Chromatographic peak integration was performed with Targetlynx software (version 4.1 SCN810, Waters Corp). Student’s *t* test was used to assess statistical significance between control and *CBS*-deficient groups for homocysteine, SAM, SAH, and methionine measurements.

Optimized compound-dependent MS parameters using Xevo TQ-S mass spectrometer

**Table:** 

Analyte	Precursor ion mass (m/z)	Fragment ion mass (m/z)	Dwell time (s)	Cone voltage (V)	Collision energy (V)
Homocysteine	136.0	90.1	0.025	15	16
SAM	399.2	136.2	0.025	62	42
SAH	385.1	134.0	0.025	66	33
Methionine	150.1	104.7	0.025	40	15

### H_2_S synthesis enzyme activity

The cellular H_2_S production rate was measured following previously described fluorescent methods with minor modifications [77]. Briefly, cultured cells were homogenized in an ice-cold buffer (50 mM Tris-HCl, pH 8.0, 50 mM NaCl) by passing through a 29G-needle syringe 20 times, followed by centrifugation at 14,000*g* for 15 min at 4 °C. The protein concentration was determined by Nanodrop with correction to remove DNA/RNA contamination. The reaction mixture contained cell extract, 50 mM Tris-HCl (Sigma-Aldrich Corp.), 1 mM L-cysteine (Sigma- Aldrich Corp.), 1 mM homocysteine (Sigma-Aldrich Corp.), 50 μM pyridoxal-5′-phosphate (MP Biomedicals, Santa Ana, CA, USA), and 10 μM H2S probe 7-azido-4-methylcoumarin (Santa Cruz Biotechnology, Santa Cruz, CA, USA). Reactions were incubated for 2 h at 37 °C and fluorescence was measured using ex 365 nm, em 450 nm with Varioskan Flash microplate reader. A linear standard curve was generated from reactions with sodium hydrosulfide.

### Methylation array profiling and analysis

GES1 parental, CRISPR control cells (P8, P17, P21, P26, and P31), and *CBS*-deficient clone 1 (P8, P15, P19, P24, and P29) and clone 2 (P8, P14, P18, P23, and P28) were cultured and harvested together at indicated time points. HFE145 parental, CRISPR control cells (P5 and P23), and *CBS*-deficient clone 1 (P5 and P23) and clone 2 (P5 and P23) were cultured and harvested together at indicated time points. Genomic DNA was hybridized on Infinium MethylationEPIC Beadchip according to the manufacturer’s specifications. Array normalization was performed using the preprocessFunnorm function from the minfi package [10.1093/bioinformatics/btw691]. Probes where the *β*-value consistently deviated more than 0.2 compared to both the CRISPR negative control and parental cell line of the same passage were considered differentially methylated. Since the global methylation analysis of the *CBS*-deficient cell lines were across all CpG contexts, we adopted a more stringent *β*-value difference of ≥ 0.2. Enrichment analysis for PRC2 related-factors was performed using ReMap 2018 v 1.2 [78]. Gastric CIMP- and EBV-CIMP-specific methylation patterns were analyzed using TCGA Infinium HumanMethylation450 beadchip data, comparing samples in either CIMP subgroup to non-CIMP samples in the same dataset. Comparison of GC methylation subtypes (gastric CIMP, EBV-CIMP, non-CIMP) to GIM methylation subtypes (“Methylation high” or CIMP, “Methylation low”’ or non-CIMP as previously published [15]) were performed at the CGI level. For this analysis, we chose a more relaxed *β*-value difference ≥ 0.1 as we were comparing at the CGI level, which is usually devoid of methylation. In the CpG context analysis, proportion of probes represented the number of hyper- or hypomethylated CpG sites divided by total number of hyper- or hypomethylated CpG sites, respectively in each sample group. Overlap between DNA methylation changes in *CBS*-deficient cells with DNA methylation changes observed in TCGA gastric CIMP and EBV-CIMP tumors was performed on CGI level, considering any CGIs that were affected on the probe level in both groups. For the pan-cancer analysis, *CBS* promoter methylation was calculated as an average of 7 CpG probes in the promoter CGI based on TCGA Infinium HumanMethylation450 beadchip data accessed through MEXPRESS [72]. CIMP groups were identified based on TCGA defined clusters (LIHC, ESCA, BLCA, THYM, HNSC) or from Karpinski et al. [52] for UCEC. Differential methylation analysis of CIMP vs non-CIMP groups, and linear regression to gene expression was performed using Wilcoxon rank sum test, and Spearman’s correlation analysis, respectively.

### *Tg-hCBS Cbs*^−/−^ mouse work

*Tg-hCBS Cbs*^*−/−*^ mice were generated as previously described [79], which lack the endogenous mouse *Cbs* gene but have a wild-type human *CBS*-encoding cDNA under the control of the Zinc-inducible metallothionein promoter. *Tg-hCBS Cbs*^−/−^ offspring (6 males and 5 females; no zinc supplementation) was obtained from an incross of *Tg-hCBS Cbs*^*−/−*^ x *Tg-hCBS Cbs*^*−/−*^, and euthanized for tissue collection at different ages (ranging from 2.1–11.1 months). Mouse stomachs were opened along the greater curvature, washed 3× in cold 1× PBS-10% FBS solution, fixed in 4% formaldehyde and embedded in paraffin for hematoxylin and eosin staining and/or RNA extraction (Qiagen RNeasy FFPE Kit). *Cbs*^*+/+*^mouse stomachs (3 males and 3 females; ranging from 2.8 to 8.1 months) from C57BL/6J mice (genetic background of *Tg-hCBS Cbs*^*−/−*^ mice) were purchased from *InVivos*, Singapore, and processed alongside the *Tg-hCBS Cbs*^−/−^ stomachs.

## Supplementary Information

**Additional file 1.** Supplementary figures.

**Additional file 2: Table S1.** List of hypermethylated gene promoters in the discovery set of CIMP vs non-CIMP cell lines identified by MeDIP-sequencing (*P* < 0.05).

**Additional file 3: Table S2.** List of downregulated genes at RNA level in the discovery set of CIMP vs non-CIMP cell lines (*P* < 0.05).

**Additional file 4: Table S3.** List of downregulated genes at protein level in the discovery set of CIMP vs non-CIMP cell lines (*P* < 0.05).

**Additional file 5: Table S4.** Protein data (LFQ intensities) for the discovery set of CIMP and non-CIMP cell lines.

**Additional file 6: Table S5.** Recurrence analysis of 6 candidate epimutations in expanded GC cell line cohort.

**Additional file 7: Table S6.** *CBS* promoter methylation levels and gene expression in expanded GC cell line cohort.

**Additional file 8: Table S7.** Overlap of genes with promoter hypermethylation (β-value difference ≥ 0.3), and significant downregulation at the RNA (*q*-value < 0.05) level in CIMP compared to non-CIMP tumours in TCGA and Singapore cohorts.

**Additional file 9: Table S8.** *CBS* germline and somatic genetic mutations in the gastric TCGA cohort.

**Additional file 10: Table S9.** List of CpG islands comprising 'GIM-GC common CIMP signature'.

**Additional file 11: Table S10.** List of differentially methylated (β-value difference ≥ 0.2) conserved CpG probes in GES1 *CBS*-deficient clones.

**Additional file 12: Table S11.** List of 76 genes with hypermethylated CpG sites (β-value difference ≥ 0.2) and downregulation (< 0.6 FD) in both GES1 *CBS*-deficient clones.

**Additional file 13: Table S12.** List of differentially methylated (β-value difference ≥ 0.2) conserved CpG probes in HFE145 *CBS*-deficient clones.

**Additional file 14: Table S13.** List of 29 genes with hypermethylated CpG sites (β-value difference ≥ 0.2) and downregulation (< 0.6 FD) in both HFE145 *CBS*-deficient clones.

**Additional file 15: Table S14.** List of CpG islands overlapping hypermethylated CpG sites in *CBS*-deficient cells and GIM-GC common CIMP signature (*P* < 1 × 10-4, bootstrapping using 10000 samples).

**Additional file 16: Table S15.** List of metabolites profiled in GES1 and HFE145 *CBS*-deficient cells.

**Additional file 17: Table S16.** Overlap of genes in GES1 *CBS*-deficient clones with MSigDB hallmark gene sets (*q*-value < 0.01).

**Additional file 18: Table S17.** Overlap of genes in HFE145 *CBS*-deficient clones with MSigDB hallmark gene sets (*q*-value < 0.01).

**Additional file 19: Table S18.** Overlap of negatively and positively correlated genes to *CBS* in gastric TCGA (*q*-value < 0.05) with MSigDB hallmark gene sets (*q*-value < 0.01).

**Additional file 20: Table S19.** Overlap of negatively correlated genes to *CBS* in various TCGA cancers (*q*-value < 0.05) with MSigDB hallmark gene sets (*q*-value < 0.01).

**Additional file 21: Table S20.** Overlap of negatively correlated genes to *CBS* in CCLE (*q*-value < 0.05) with MSigDB hallmark gene sets (*q*-value < 0.01).

**Additional file 22: Table S21.** Gene expression analysis of *Cbs*+/+ vs. *Cbs*-/- mice.

**Additional file 23: Table S22.** List of upregulated genes and pathways in CBS-deficient mouse stomachs (FDR < 0.05).

**Additional file 24: Table S23.** Top 20 pathways in the MSigDB overlap (*q*-value < 0.01) of differentially expressed genes in *CBS*-deficient mouse gastric tissue.

**Additional file 25.** Review history.

## Data Availability

The sequencing data generated from the study can be accessed at NCBI SRA (PRJNA628586) [80]. The methylation array data generated from the study can be accessed at NCBI GEO (GSE149411) [81]. The H3K27me3 ChIP data was accessed from NCBI GEO GSE121140 [82]. DNA methylation and gene expression data for all TCGA datasets were obtained from MEXPRESS [83]. DNA methylation and gene expression data for SG cohort was accessed from NCBI GEO GSE30601 [4] and GSE15460 respectively [84]. DNA methylation data for GIM was accessed from NCBI GEO GSE103186 [15]. *CBS* methylation data for BE was accessed from Krause et al. (Additional file 5, Supplementary Table 4) [18]. Infinium HumanMethylation450 beadchip array data, gene expression, and mutational status of select cancer genes for the GC cell lines were accessed through the Singapore Gastric Cancer Consortium (http://www.sgcc.sg/research/unique-resources). Other datasets supporting the conclusions of this article are included within the article as additional files. All other datasets used and/or analyzed during the current study are available from the corresponding author on reasonable request.
