# Author Correction: Highly recurrent *CBS* epimutations in gastric cancer CpG island methylator phenotypes and inflammation

**DOI:** 10.1186/s13059-021-02405-z

**Published:** 2021-06-17

**Authors:** Nisha Padmanabhan, Huang Kie Kyon, Arnoud Boot, Kevin Lim, Supriya Srivastava, Shuwen Chen, Zhiyuan Wu, Hyung-Ok Lee, Vineeth T. Mukundan, Charlene Chan, Yarn Kit Chan, Ong Xuewen, Jason J. Pitt, Zul Fazreen Adam Isa, Manjie Xing, Ming Hui Lee, Angie Lay Keng Tan, Shamaine Ho Wei Ting, Micah A. Luftig, Dennis Kappei, Warren D. Kruger, Jinsong Bian, Ying Swan Ho, Ming Teh, Steve George Rozen, Patrick Tan

**Affiliations:** 1 Programme in Cancer and Stem Cell Biology, Duke-NUS Medical School, 8, College road, Singapore, 169857 Singapore; 2 Centre for Computational Biology, Duke-NUS Medical School, Singapore, 169857 Singapore; 3 Department of Medicine, Yong Loo Lin School of Medicine, National University of Singapore, Singapore, 119228 Singapore; 4 Bioprocessing Technology Institute, A*STAR, 20 Biopolis Way, #06-01 Centros, Singapore, 138668 Singapore; 5 Department of Pharmacology, Yong Loo Lin School of Medicine, National University of Singapore, Singapore, 117600 Singapore; 6 Cancer Biology Program, Fox Chase Cancer Center, Philadelphia, PA USA; 7 Cancer Science Institute of Singapore, National University of Singapore, Singapore, 117599 Singapore; 8 Department of Molecular Genetics and Microbiology, Duke Centre for Virology, Duke University School of Medicine, Durham, NC USA; 9 Department of Biochemistry, Yong Loo Lin School of Medicine, National University of Singapore, Singapore, 117596 Singapore; 10 National University of Singapore (Suzhou) Research Institute, Suzhou, 215123 China; 11 Department of Pathology, National University of Singapore, Singapore, 119228 Singapore; 12 Genome Institute of Singapore, Singapore, 138672 Singapore; 13 SingHealth/Duke-NUS Institute of Precision Medicine, National Heart Centre Singapore, Singapore, 169856 Singapore; 14 Singapore Gastric Cancer Consortium, Singapore, 119074 Singapore; 15 Department of Physiology, National University of Singapore, Singapore, 117593 Singapore

**Correction to: Genome Biol 22, 167 (2021)**

**https://doi.org/10.1186/s13059-021-02375-2**

Following publication of the original article [1], the authors identified an error in the author name of Hyung-Ok Lee.

The incorrect author name is: Hyung-O K Lee

The correct author name is: Hyung-Ok Lee

The author group has been updated above and the original article [1] has been corrected.

## References

[1] Padmanabhan N, Kyon HK, Boot A (2021). Highly recurrent *CBS* epimutations in gastric cancer CpG island methylator phenotypes and inflammation. Genome Biol.

