# Supplementary material for: Highly recurrent CBS epimutations in gastric cancer CpG island methylator phenotypes and inflammation
Source: Genome Biol. 2021 Jun 1;22:167. doi: 10.1186/s13059-021-02375-2 (PMC8170989; doi:10.1186/s13059-021-02375-2)
Supplement: Supplementary file 1 — Additional file 1. Supplementary figures. [file 13059_2021_2375_MOESM1_ESM.pdf]

Fig. S1. Subtyping of GC cell lines to CIMP clusters. Unsupervised hierarchical clustering of 62 GC cell lines based on the top 10,000 most variable CGI probes identified 4 major branches, that was categorized into 3 distinct clusters, designated as 'CIMP-high' (branch 1), 'CIMP-low' (branches 3, 4) and 'non-CIMP' (branch 2). EBV, MSI and mutational status of select cancer genes in each cluster are also indicated. Colour key represents methylation β-values.

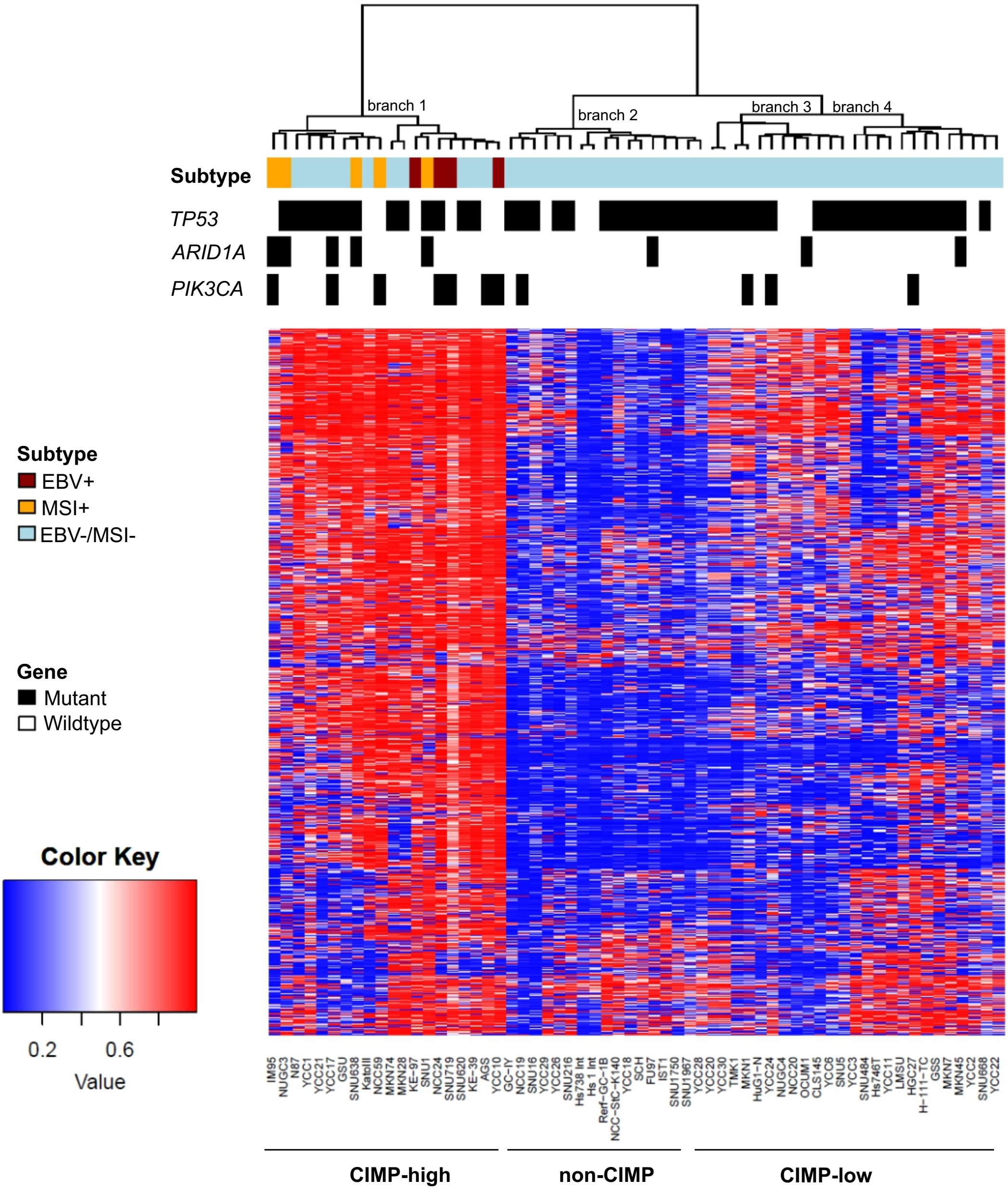
branch 1
branch 2
branch 3
branch 4
Subtype
TP53
ARID1A
PIK3CA
Subtype
EBV+
MSI+
EBV-/MSI-
Gene
Mutant
Wildtype
Color Key
0.2
0.6
Value
IM95
NUGC3
N87
YCC1
YCC21
YCC17
GSU
SNU638
KatoIII
NCC59
MKN74
MKN28
KE-97
SNU1
NCC24
SNU719
SNU620
KE-39
AGS
YCC10
GC-IY
NCC19
SNU16
YCC29
YCC26
SNU216
Hs738 Int
Hs 1 Int
Rerf-GC-1B
NCC-StC-K140
YCC18
SCH
FU97
IST1
SNU1750
SNU1967
YCC28
YCC20
YCC30
TMK1
MKN1
HuG1-N
YCC24
NUGC4
NCC20
OCUM1
CLS145
YCC6
SNU5
YCC3
SNU484
Hs746T
YCC11
LMSU
HGC27
H-111-TC
GSS
MKN7
MKN45
YCC2
SNU668
YCC22
CIMP-high
non-CIMP
CIMP-low

Fig. S2. Validation of *CBS* RNA and protein levels. (A) *CBS* mRNA expression in normal gastric cell lines [n=2] (Average Delta Ct = 6.8 - 7.0), CIMP lines [n=6] (Average Delta Ct = 16.4), and non-CIMP lines [n=6] (Average Delta Ct = 7.5 - 8.5) assayed by quantitative PCR (**B**) Protein levels of CBS in SNU1750 cell line treated with non-targeting or *CBS* targeted siRNAs [top panel] with knockdown efficiency validated using quantitative PCR [bottom panel] (n=3) (*P<0.05) (**C**) *CBS* mRNA expression in 24 normal human stomachs assayed by quantitative PCR (Average Delta Ct = 7.6) (**D**) Immunohistochemistry of CBS antibody in human tissue with high CBS mRNA expression, liver [top panel] and low CBS mRNA expression, thigh muscle [bottom panel] according to GTEx portal [Mean TPM of 135.9 in liver and 0.8 in skeletal muscle]. Control sections were not treated with the primary antibody. TPM, Transcripts Per Million; kDa, kilodalton; ND, Not determined.

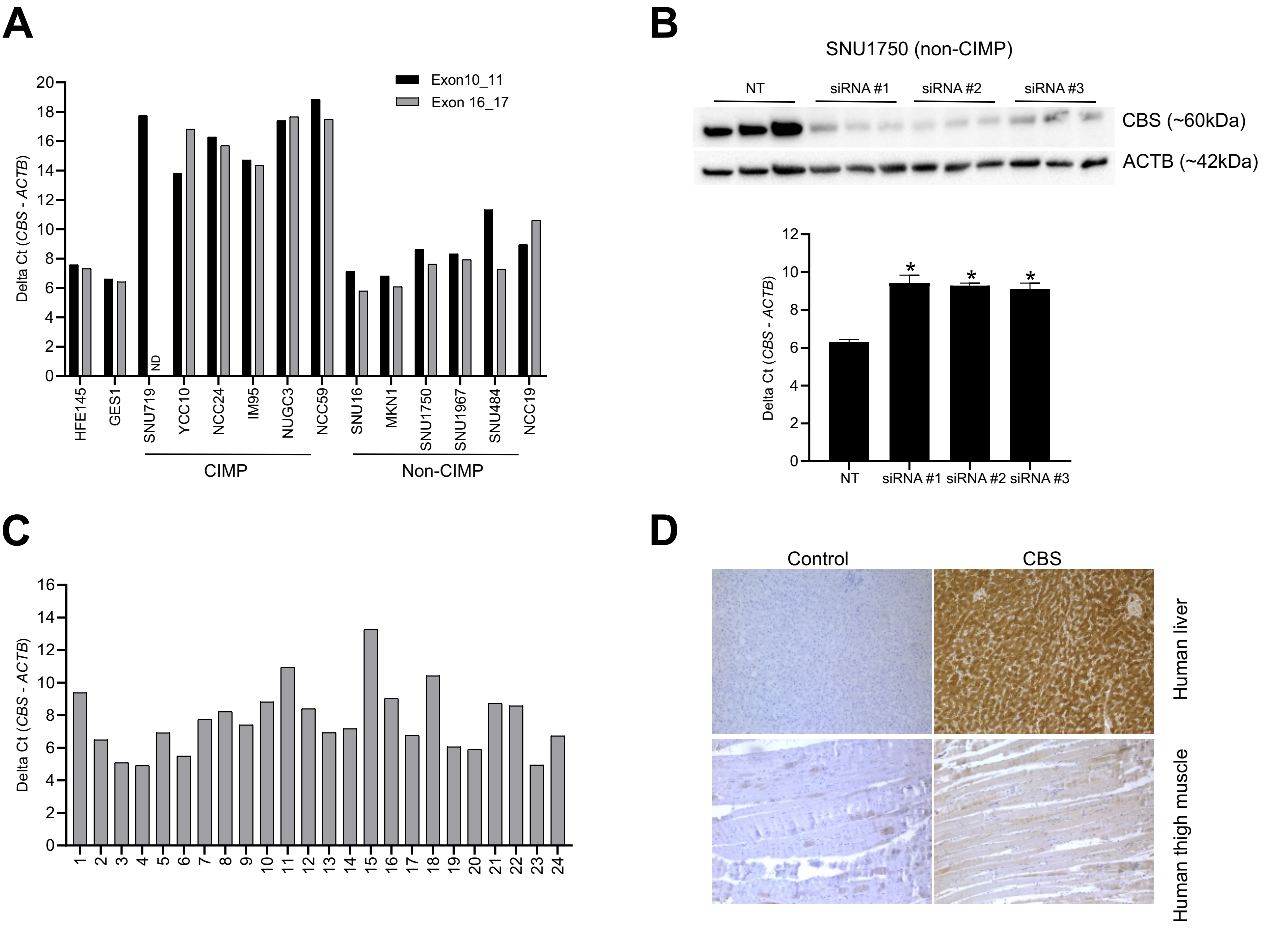

A
B
C
D
Exon10_11
Exon 16_17
Delta Ct (CBS - ACTB)
ND
HFE145
GES1
SNU719
YCC10
NCC24
IM95
NUGC3
NCC59
SNU16
MKN1
SNU1750
SNU1967
SNU484
NCC19
CIMP
Non-CIMP
SNU1750 (non-CIMP)
NT
siRNA #1
siRNA #2
siRNA #3
CBS (~60kDa)
ACTB (~42kDa)
Control
CBS
Human liver
Human thigh muscle

Fig. S3. Uncropped western blotting images. CBS protein levels in **(A)** Normal gastric, CIMP, and non-CIMP GC lines as shown in Figure 1D (**B**) SNU1750 cell line treated with non-targeting or *CBS* targeted siRNAs as shown in Supplementary Figure 2B (**C**) GES1 CRISPR control and *CBS*-deficient CRISPR clones as shown in Figure 4B (**D**) HFE145 CRISPR control, *CBS*-deficient CRISPR clone 1 and clone 2 cells as shown in Figure S9A.

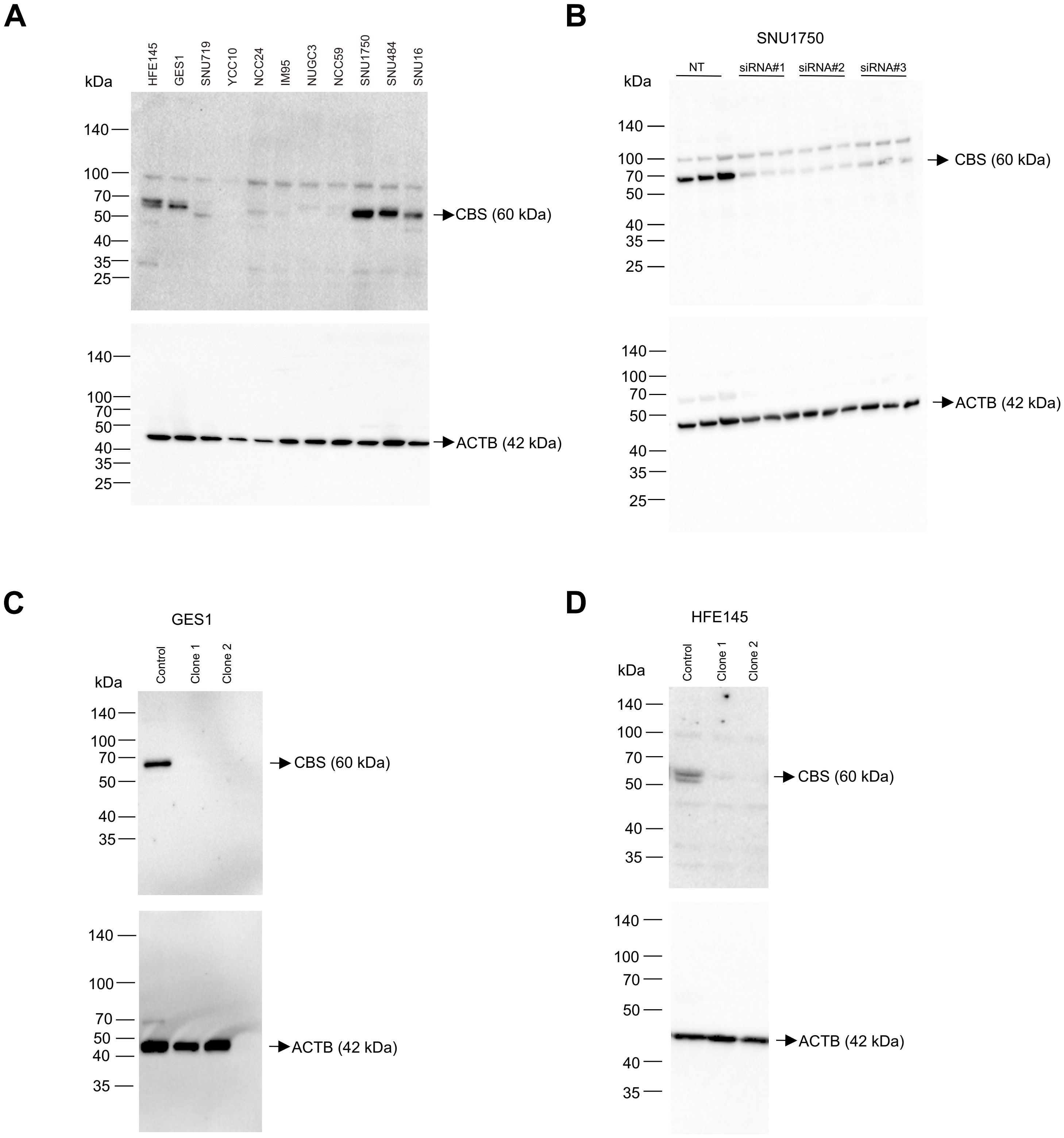
A
kDa
HFE145
GES1
SNU719
YCC10
NCC24
IM95
NUGC3
NCC59
SNU1750
SNU484
SNU16
140
100
70
50
40
35
25
CBS (60 kDa)
ACTB (42 kDa)
B
SNU1750
NT
siRNA#1
siRNA#2
siRNA#3
CBS (60 kDa)
ACTB (42 kDa)
C
GES1
Control
Clone 1
Clone 2
CBS (60 kDa)
ACTB (42 kDa)
D
HFE145
Control
Clone 1
Clone 2
CBS (60 kDa)
ACTB (42 kDa)

Fig. S4. Lack of H3K27me3 enrichment at *CBS* promoter in GC CIMP cell lines. (A) Representative example showing enrichment of 5-methyl cytosine [5-mc] at *CBS* promoter in CIMP cell line IM95 (upper panel), and lack of enrichment of H3K27me3 in CIMP cell lines IM95 and AGS (lower panel) in the same region. SNU484 and YCC11 are non-CIMP cell lines. Y-axis for 5-mc tracks represents enrichment over input. Y-axis for H3K27me3 tracks represents reads per kilobase million. **(B**) Positive controls for H3K27me3-ChIP showing enrichment at *ZRANB2-AS1* in AGS, IM95, SNU484 and YCC11 cell lines (left panel) and *LHFPL4* (right panel) in AGS, IM95, and YCC11 cell lines.

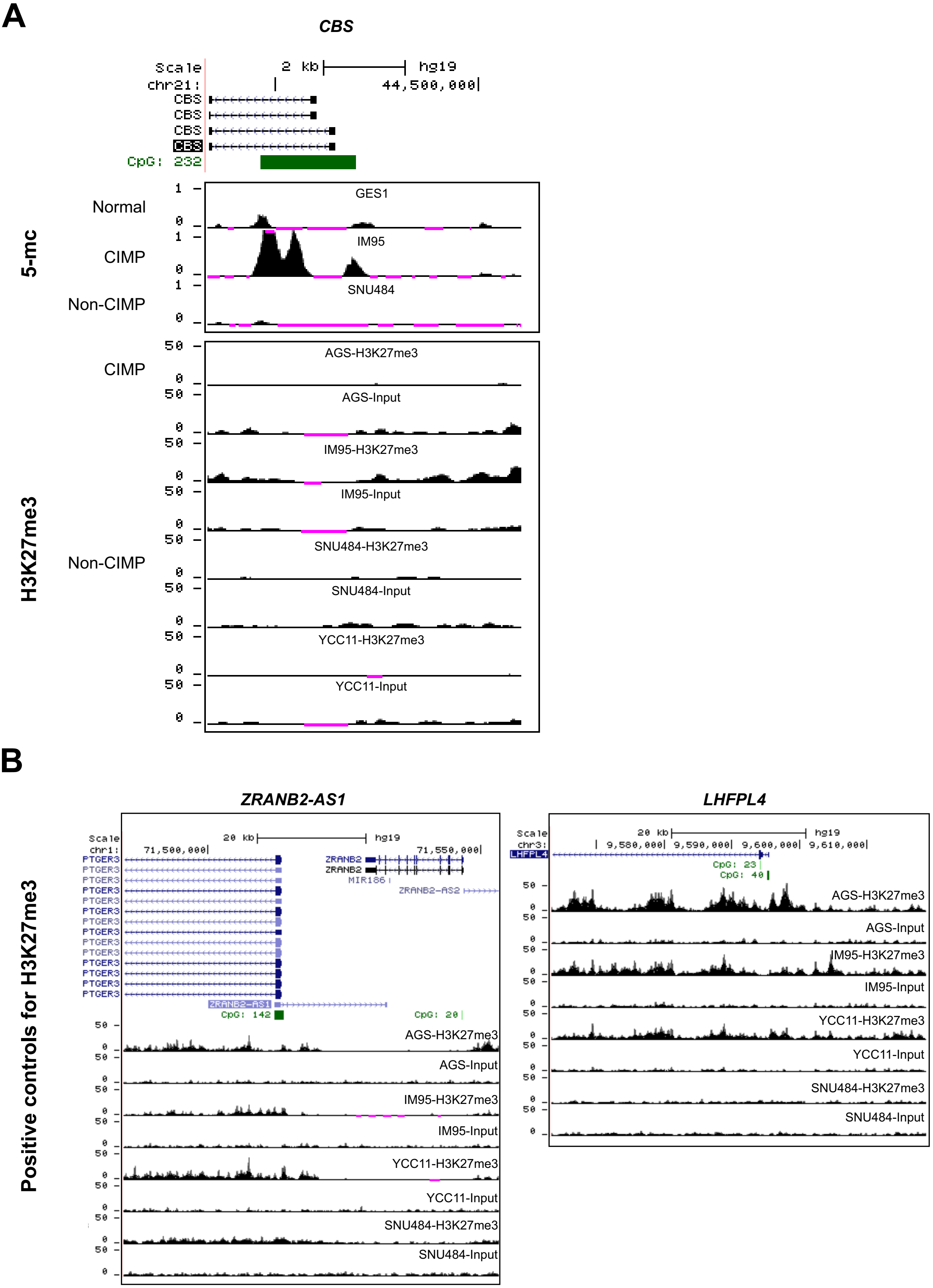
A
CBS
Scale
2 kb
hg19
chr21:
44,500,000
CBS
CBS
CBS
CBS
CpG: 232
5-mc
Normal
CIMP
Non-CIMP
GES1
IM95
SNU484
H3K27me3
CIMP
Non-CIMP
AGS-H3K27me3
AGS-Input
IM95-H3K27me3
IM95-Input
SNU484-H3K27me3
SNU484-Input
YCC11-H3K27me3
YCC11-Input
B
Positive controls for H3K27me3
ZRANB2-AS1
Scale
20 kb
hg19
chr1:
71,500,000
71,550,000
PTGER3
ZRANB2
ZRANB2
MIR186
ZRANB2-AS2
ZRANB2-AS1
CpG: 142
CpG: 20
AGS-H3K27me3
AGS-Input
IM95-H3K27me3
IM95-Input
YCC11-H3K27me3
YCC11-Input
SNU484-H3K27me3
SNU484-Input
LHFPL4
Scale
20 kb
hg19
chr3:
9,580,000
9,590,000
9,600,000
9,610,000
LHFPL4
CpG: 23
CpG: 40
AGS-H3K27me3
AGS-Input
IM95-H3K27me3
IM95-Input
YCC11-H3K27me3
YCC11-Input
SNU484-H3K27me3
SNU484-Input

Fig. S5. Distribution of *CBS* promoter methylation values in CIMP and non-CIMP categories in multiple tumours. Graph representing the percentage of CIMP and non-CIMP samples in each tumour type harbouring different levels of *CBS* promoter methylation levels based on β-values. CIMP tumours tend to carry higher *CBS* promoter β-values vs. non-CIMP tumours (BLCA: 63.8% vs. 17.1%, UCEC: 29.3% vs. 1.80%, EAC: 93.0% vs. 37.90%, HNSC: 76.7% vs. 17.7%, LIHC: 28.8% vs. 15.6%, STAD: 98.8% vs. 49.2%).

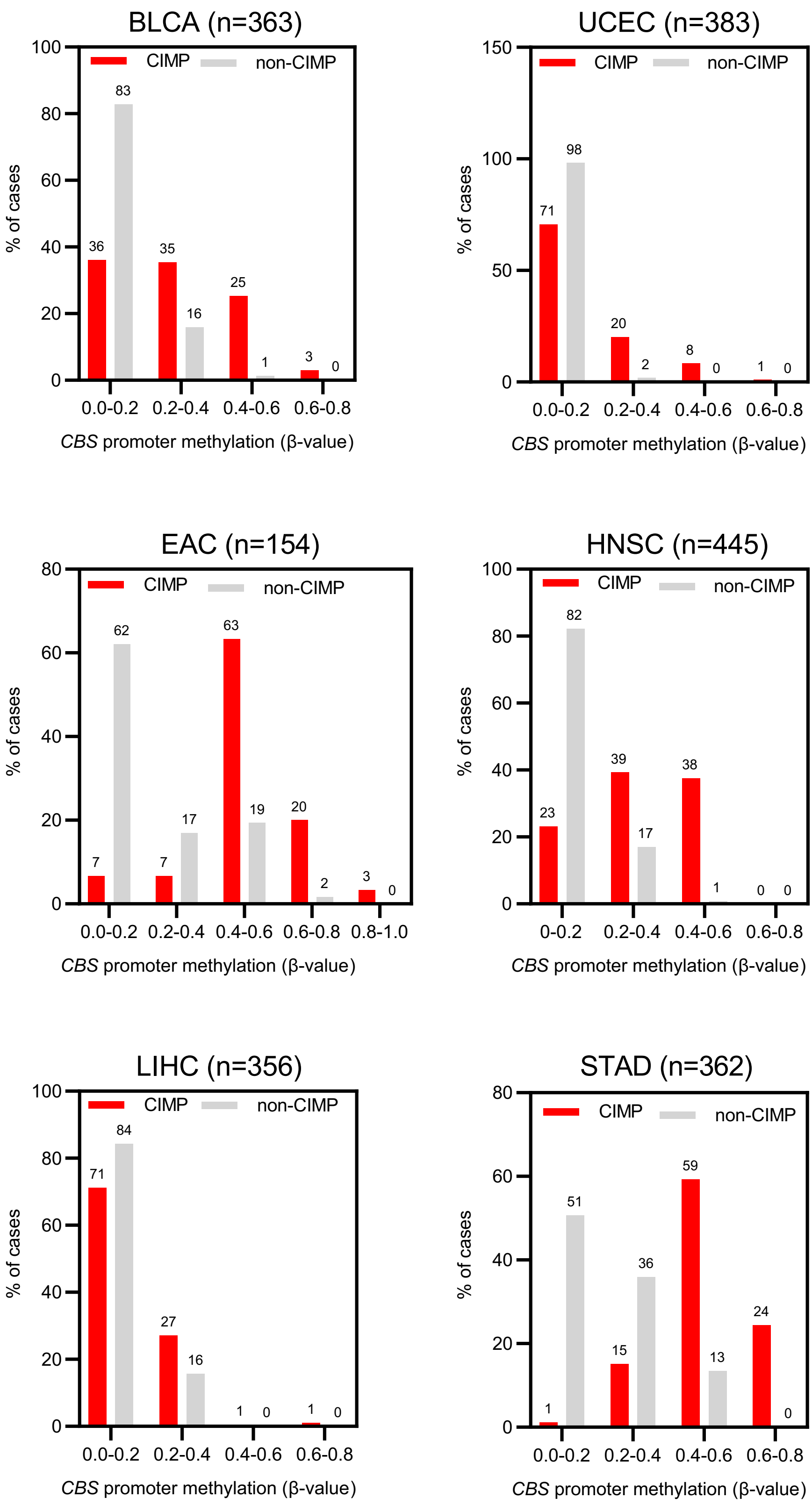
BLCA (n=363)
UCEC (n=383)
EAC (n=154)
HNSC (n=445)
LIHC (n=356)
STAD (n=362)
CIMP
non-CIMP
% of cases
CBS promoter methylation (β-value)
0.0-0.2
0.2-0.4
0.4-0.6
0.6-0.8
0.8-1.0
0-0.2
36
83
35
16
25
1
3
0
71
98
20
2
8
0
1
0
7
62
7
17
63
19
20
2
3
0
23
82
39
17
38
1
0
0
71
84
27
16
1
0
1
0
1
51
15
36
59
13
24
0

Fig. S6. Correlation plots of *CBS* gene expression and promoter methylation in multiple cancers. Correlation plots of *CBS* gene expression (RSEM) and DNA methylation (β-values) in BLCA, EAC, HNSC, LIHC, UCEC and STAD. Spearman r and P-values are indicated per cancer type.

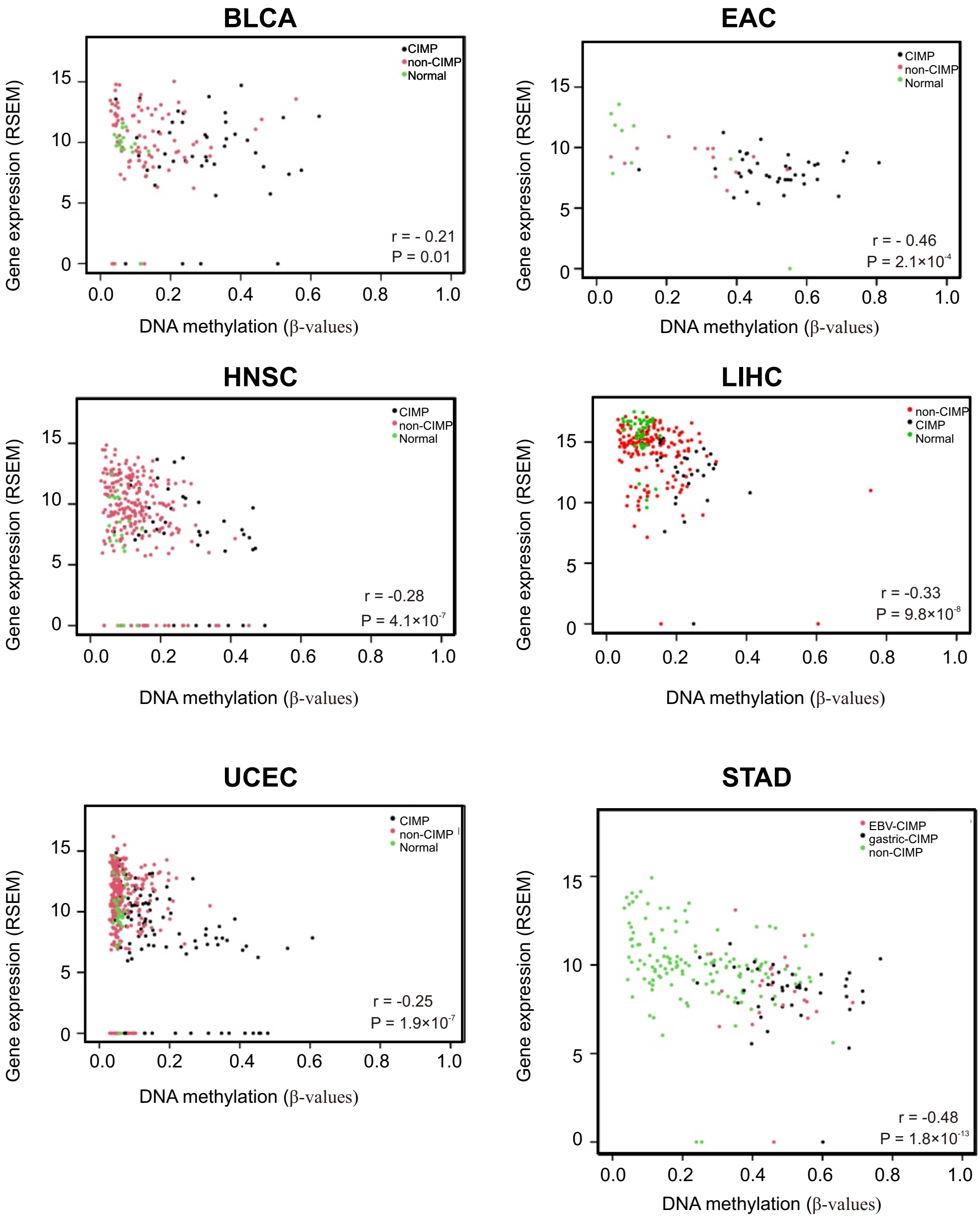

BLCA
CIMP
non-CIMP
Normal
r = - 0.21
P = 0.01
Gene expression (RSEM)
DNA methylation (β-values)
EAC
CIMP
non-CIMP
Normal
r = - 0.46
P = 2.1×10-4
Gene expression (RSEM)
DNA methylation (β-values)
HNSC
CIMP
non-CIMP
Normal
r = -0.28
P = 4.1×10-7
Gene expression (RSEM)
DNA methylation (β-values)
LIHC
non-CIMP
CIMP
Normal
r = -0.33
P = 9.8×10-8
Gene expression (RSEM)
DNA methylation (β-values)
UCEC
CIMP
non-CIMP
Normal
r = -0.25
P = 1.9×10-7
Gene expression (RSEM)
DNA methylation (β-values)
STAD
EBV-CIMP
gastric-CIMP
non-CIMP
r = -0.48
P = 1.8×10-13
Gene expression (RSEM)
DNA methylation (β-values)

Fig. S7. ***CBS*** **promoter is hypermethylated in Barret's oesophagus.** Heat map representing mean methylation β-values of 14 CpG probes in the 5'UTR and TSS region of *CBS* gene in normal, GERD [gastroesophageal reflux disease], NSE [non-tumour squamous oesophagus], BE [Barret's oesophagus], and EAC [Oesophageal adenocarcinoma] groups reported as significant (P<0.05) by Krause et al [18].

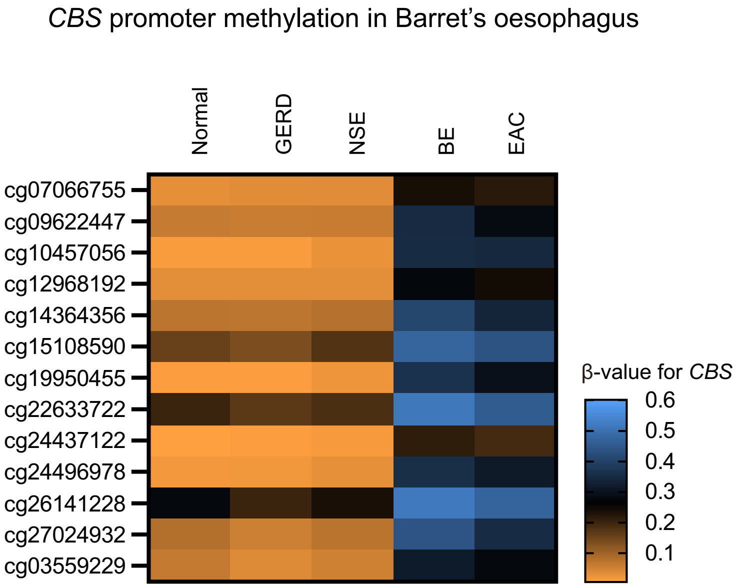
CBS promoter methylation in Barret's oesophagus
Normal
GERD
NSE
BE
EAC
cg07066755
cg09622447
cg10457056
cg12968192
cg14364356
cg15108590
cg19950455
cg22633722
cg24437122
cg24496978
cg26141228
cg27024932
cg03559229
β-value for CBS
0.6
0.5
0.4
0.3
0.2
0.1

Fig. S8. Venn diagram of differentially methylated probes in random pairing of GES1, parental, CRISPR control and *CBS*-deficient cells. Differential methylation analysis of random pairing of samples (β-value difference ≥ 0.2) across passages indicated no conserved signature.

(Clone 2 and Parental)
vs.
(Clone 1 and CRISPR control)

(Clone 1 and Parental)
vs.
(Clone 2 and CRISPR control)

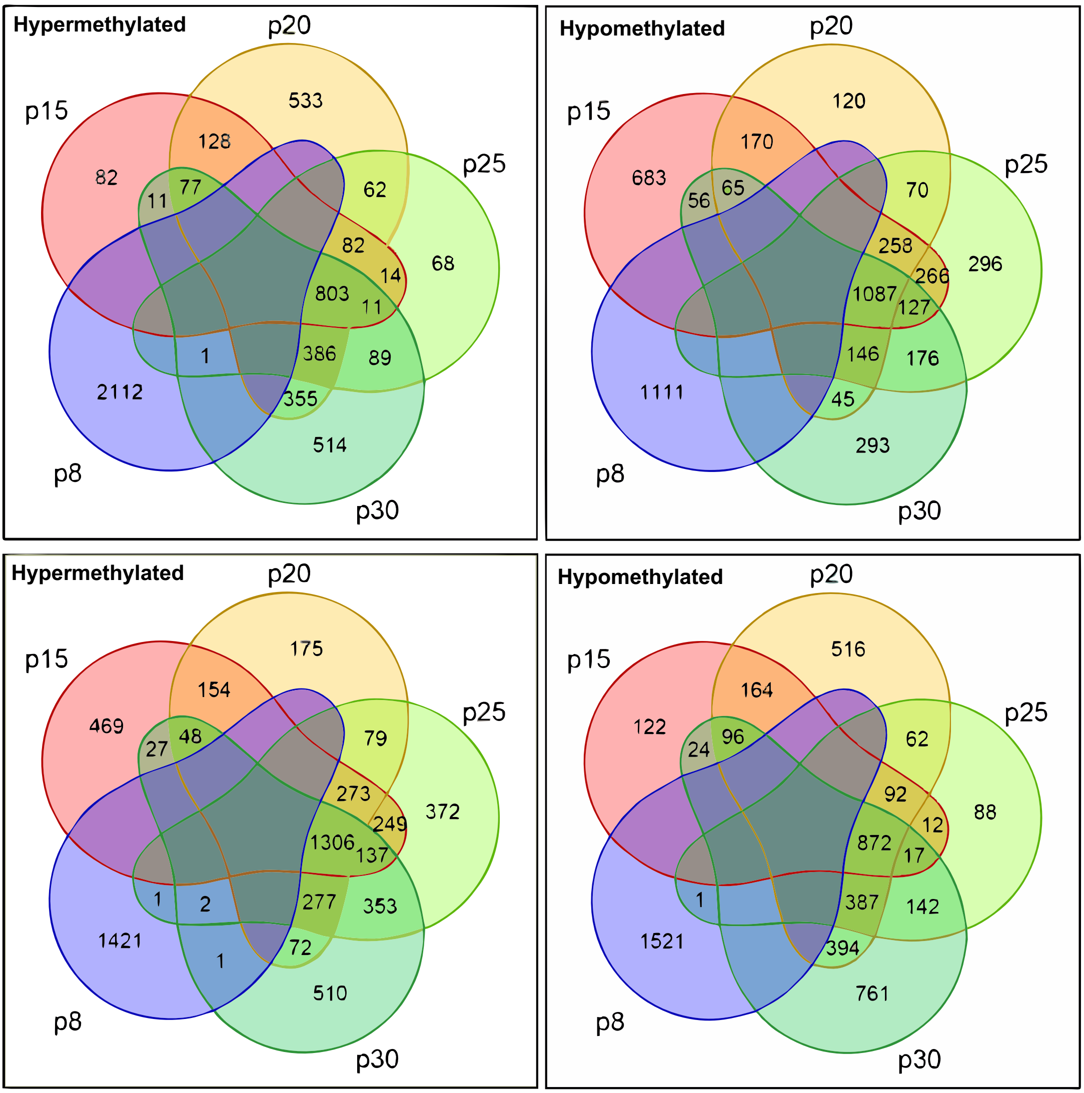

Fig. S9. Loss of CBS alters the DNA methylation landscape in normal gastric epithelial cell line HFE145. (**A**) CBS protein levels in HFE145 CRISPR control, *CBS*-deficient CRISPR clone 1 and clone 2 cells using Western blotting (**B**) Venn diagram of differential methylation analysis of HFE145 *CBS*-deficient clone 1 and clone 2 compared to parental and CRISPR control cells (β-value difference ≥ 0.2) between P5 and P23 (**C**) Venn diagram of differential methylation analysis of random pairing of samples (β-value difference ≥ 0.2) across 2 passages indicated minimal conserved signature (**D**) Breakdown of the HFE145 *CBS*-deficient conserved signature in CpG contexts island, shore, shelf and open sea; * $p<0.001$ according to binomial test; 'Annotated' refers to the distribution of CpG probes on the Infinium methylation EPIC array, 'Random' refers to the sum of all differentially methylated CpG probes in random pairing of samples (**E**) Unsupervised clustering of conserved hypermethylated 3241 CpG sites in HFE145 *CBS*-deficient clones separates them from parental and CRISPR control cells (**F**) Enrichment of PRC2-related factors in *CBS*-deficient conserved hypermethylation signatures. kDa, kilodalton.

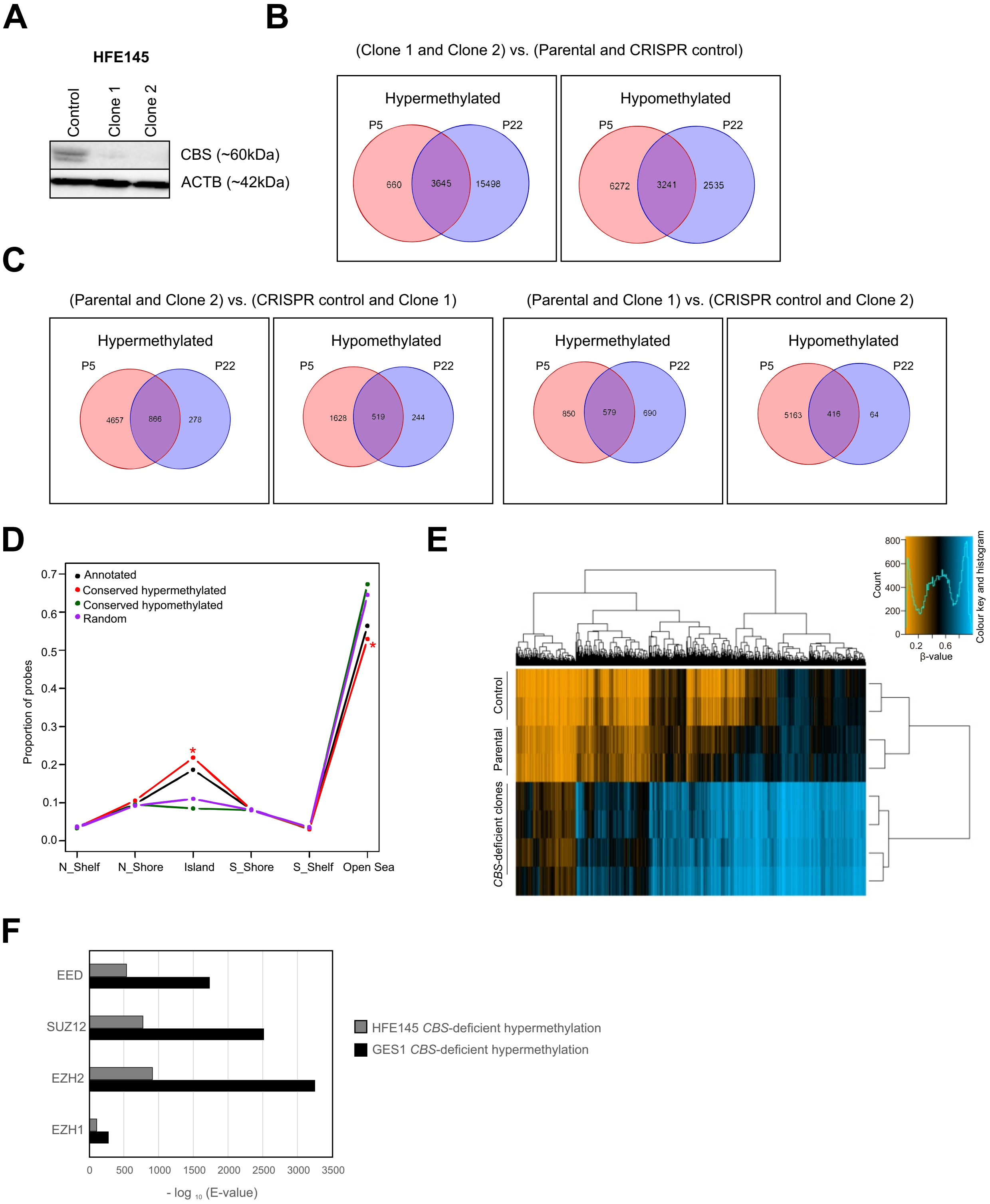
A
HFE145
Control
Clone 1
Clone 2
CBS (~60kDa)
ACTB (~42kDa)
B
(Clone 1 and Clone 2) vs. (Parental and CRISPR control)
Hypermethylated
P5
P22
660
3645
15498
Hypomethylated
P5
P22
6272
3241
2535
C
(Parental and Clone 2) vs. (CRISPR control and Clone 1)
Hypermethylated
P5
P22
4657
866
278
Hypomethylated
P5
P22
1628
519
244
(Parental and Clone 1) vs. (CRISPR control and Clone 2)
Hypermethylated
P5
P22
850
579
690
Hypomethylated
P5
P22
5163
416
64
D
Annotated
Conserved hypermethylated
Conserved hypomethylated
Random
Proportion of probes
0.0
0.1
0.2
0.3
0.4
0.5
0.6
0.7
N_Shelf
N_Shore
Island
S_Shore
S_Shelf
Open Sea
E
Count
800
600
400
200
0
0.2
0.6
β-value
Colour key and histogram
Control
Parental
CBS-deficient clones
F
EED
SUZ12
EZH2
EZH1
0
500
1000
1500
2000
2500
3000
3500
- log 10 (E-value)
HFE145 CBS-deficient hypermethylation
GES1 CBS-deficient hypermethylation

Fig. S10. *CBS*-deficiency causes altered metabolic state in normal gastric cells with significant dysregulation of lipids. (A) Pie charts representing metabolic changes in *CBS*-deficient GES1 and HFE145 cells (upper panel) and list of conserved metabolites (lower panel) (**B**) Gene expression levels (FPKM) of *NNMT* in *CBS*-deficient clones compared to controls (**C**) Heat map of significantly altered lipids (P<0.05) in *CBS*-deficient clones compared to controls; red underline indicates conserved metabolites between cell lines.

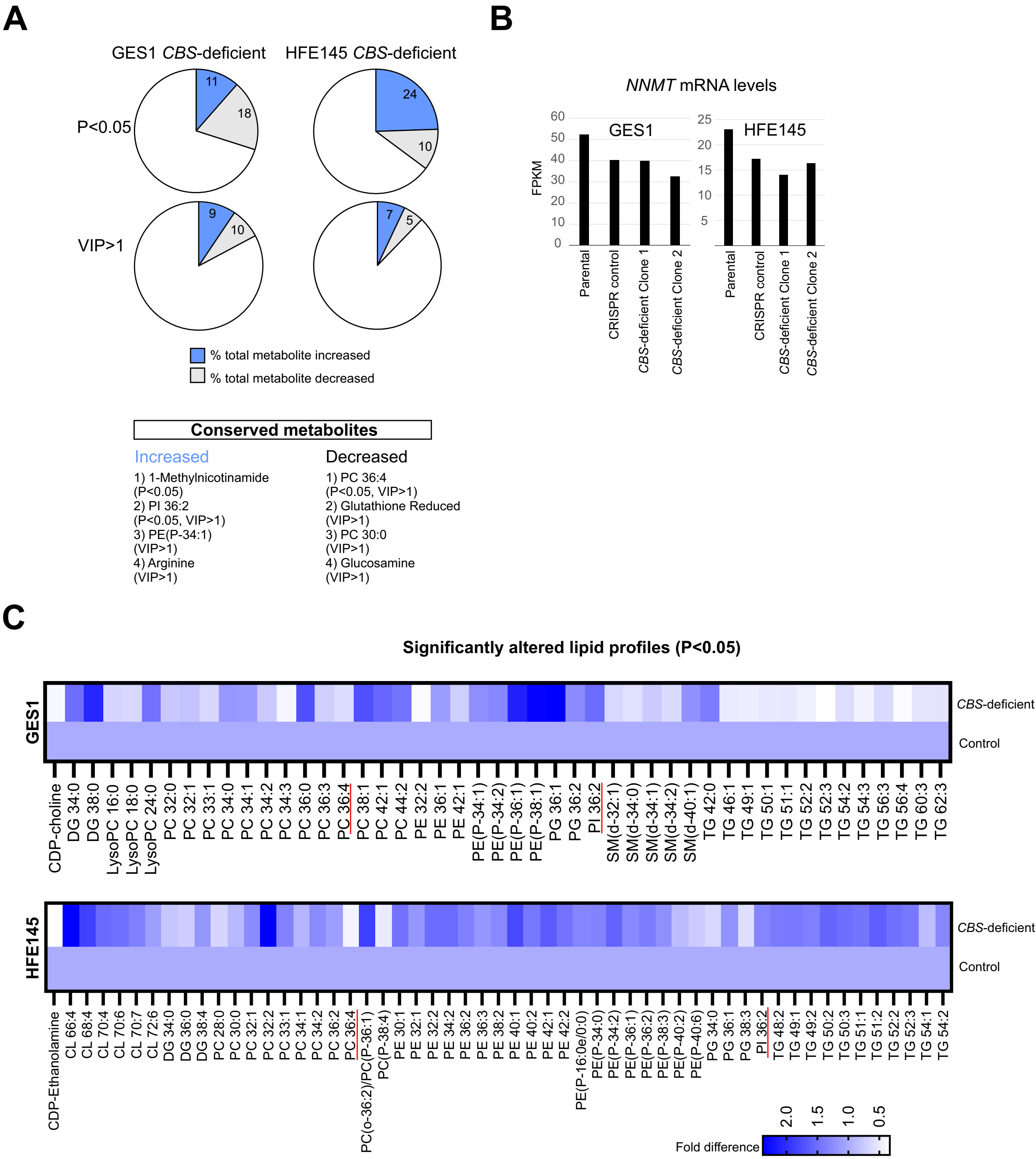
A
GES1 CBS-deficient
HFE145 CBS-deficient
P<0.05
VIP>1
11
18
24
10
9
10
7
5
% total metabolite increased
% total metabolite decreased
Conserved metabolites
Increased
1) 1-Methylnicotinamide (P<0.05)
2) PI 36:2 (P<0.05, VIP>1)
3) PE(P-34:1) (VIP>1)
4) Arginine (VIP>1)
Decreased
1) PC 36:4 (P<0.05, VIP>1)
2) Glutathione Reduced (VIP>1)
3) PC 30:0 (VIP>1)
4) Glucosamine (VIP>1)
B
NNMT mRNA levels
FPKM
GES1
HFE145
Parental
CRISPR control
CBS-deficient Clone 1
CBS-deficient Clone 2
C
Significantly altered lipid profiles (P<0.05)
GES1
CBS-deficient
Control
CDP-choline
DG 34:0
DG 38:0
LysoPC 16:0
LysoPC 18:0
LysoPC 24:0
PC 32:0
PC 32:1
PC 33:1
PC 34:0
PC 34:1
PC 34:2
PC 34:3
PC 36:0
PC 36:3
PC 36:4
PC 38:1
PC 42:1
PC 44:2
PE 32:2
PE 36:1
PE 42:1
PE(P-34:1)
PE(P-34:2)
PE(P-36:1)
PE(P-38:1)
PG 36:1
PG 36:2
PI 36:2
SM(d-32:1)
SM(d-34:0)
SM(d-34:1)
SM(d-34:2)
SM(d-40:1)
TG 42:0
TG 46:1
TG 49:1
TG 50:1
TG 51:1
TG 52:2
TG 52:3
TG 54:2
TG 54:3
TG 56:3
TG 56:4
TG 60:3
TG 62:3
HFE145
CBS-deficient
Control
CDP-Ethanolamine
CL 66:4
CL 68:4
CL 70:4
CL 70:6
CL 70:7
CL 72:6
DG 34:0
DG 36:0
DG 38:4
PC 28:0
PC 30:0
PC 32:1
PC 32:2
PC 33:1
PC 34:1
PC 34:2
PC 36:2
PC 36:4
PC(o-36:2)/PC(P-36:1)
PC(P-38:4)
PE 30:1
PE 32:1
PE 32:2
PE 34:2
PE 36:2
PE 36:3
PE 38:2
PE 40:1
PE 40:2
PE 42:1
PE 42:2
PE(P-16:0e/0:0)
PE(P-34:0)
PE(P-34:2)
PE(P-36:1)
PE(P-36:2)
PE(P-38:3)
PE(P-40:2)
PE(P-40:6)
PG 34:0
PG 36:1
PG 38:3
PI 36:2
TG 48:2
TG 49:1
TG 49:2
TG 50:2
TG 50:3
TG 51:1
TG 51:2
TG 52:2
TG 52:3
TG 54:1
TG 54:2
Fold difference
2.0
1.5
1.0
0.5

Fig. S11. Proposed model for *CBS* epimutation in GC. Epimutation at the promoter locus of *CBS* remodels normal gastric epithelial cells through simultaneous perturbation of the methionine and homocysteine cycles. This results in reshaping of the DNA methylation landscape, and an intrinsic inflammatory cellular response. Pre-malignant gastric intestinal metaplasia lesions with *CBS* hypermethylation may thus be more prone to chronic inflammation and benefit from anti-inflammatory H_2S donor therapies. *CBS* loss influences DNA methylation likely through multiple mechanisms. CIMP, CpG island methylator phenotype; EBV, Epstein - Barr virus; MSI, Microsatellite instability.

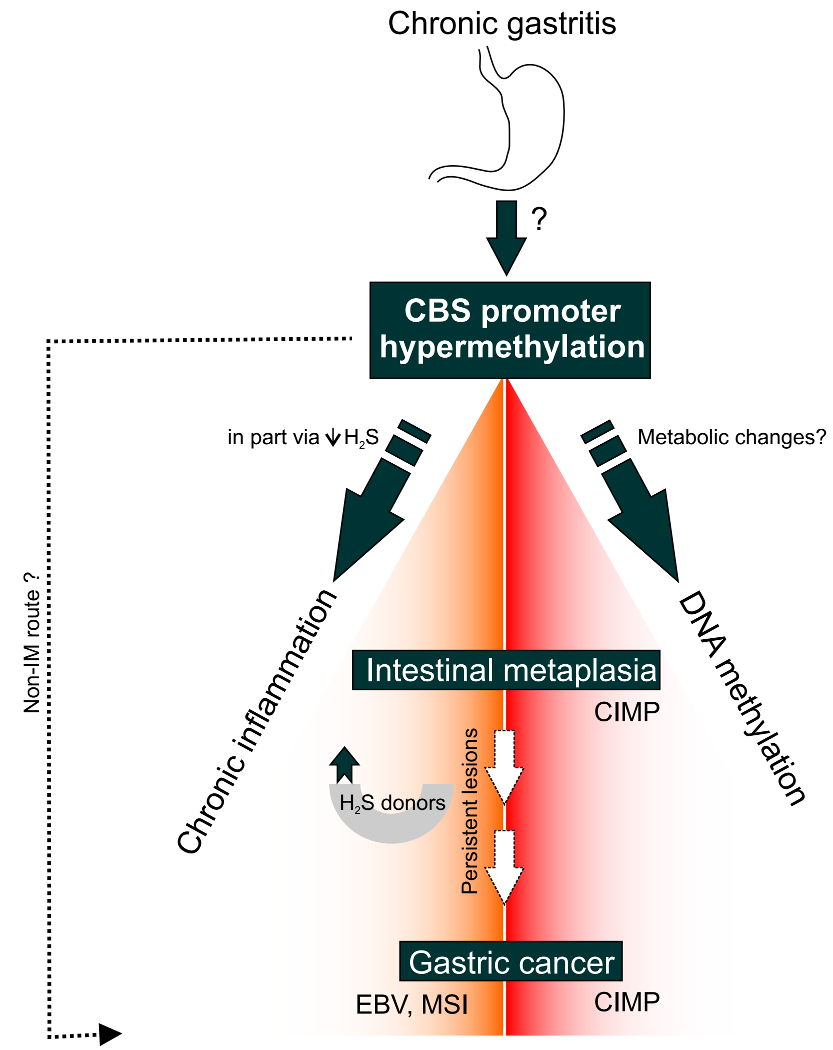

Chronic gastritis
?
CBS promoter hypermethylation
in part via ↓H_2S
Metabolic changes?
Chronic inflammation
DNA methylation
Non-IM route ?
Intestinal metaplasia
CIMP
H_2S donors
Persistent lesions
Gastric cancer
EBV, MSI
CIMP
