# Supplementary material for: Highly recurrent CBS epimutations in gastric cancer CpG island methylator phenotypes and inflammation
Source: Genome Biol. 2021 Jun 1;22:167. doi: 10.1186/s13059-021-02375-2 (PMC8170989; doi:10.1186/s13059-021-02375-2)
Supplement: Supplementary file 25 — Additional file 25. Review history. [file 13059_2021_2375_MOESM25_ESM.docx]

Review History

**First round of review**

**Reviewer 1**

**Are you able to assess all statistics in the manuscript, including the appropriateness of statistical tests used?** No, I do not feel adequately qualified to assess the statistics.

**Comments to author:**

This study clearly demonstrated a novel epigenetic alteration which could potentially lead to development of CpG island methylator phenotype-positive gastric cancer. This study is well designed, experiments were adequately performed, and results are solid to support the conclusion. I agree to accept this manuscript.

**Reviewer 2**

**Are you able to assess all statistics in the manuscript, including the appropriateness of statistical tests used?** Yes, and I have assessed the statistics in my report.

**Comments to author:**

The authors associate loss of CBS, by epigenetic silencing (epimutation), to the CIMP phenotype in gastric cancers and other cancers. Deleting CBS by CRISPR causes increased methylation resembling CIMP. Mechanistically, the authors argue that CBS loss alters methionine and homocysteine metabolism, which may cause the methylation changes. The authors show that CBS transcriptional loss as the most frequent and specific marker 50 GC CIMP-high cell lines from a panel of 50 GC cell lines. Using priary GC cancer datasets from TCGA and Singapore cohort, they show that CBS methylation/silencing associated with CIMP phenotype. About 1/3rd of all cancers are observed to have CBS downregulation in their CIMP+ counterparts. Hypermethylated CGIs in CBS deficient cells have a good 38% overlap with the commonly hypermethylated CGIs in patient GIM and GC samples.

Overall the manuscript is well presented. Mechanisms underlying CIMP are of general interest to the field. The proposed role of CBS in causing CIMP is a novel observation and deserves careful consideration and publication. Following are some concerns that need to be addressed.

-	Mechanistic link to activation of inflammatrory pathway by reduction in H2S levels is not clear. In the in vivo experiments with CbS-/- mice, the inflammatory pathways identified are not the same as that identified in cell lines data sets (5E, F). Thus, the impact of CBS on activating inflammatory response, should be carefully interpreted/stated in the paper, with the proper caveats to this interpretation given that the data is not conclusive. Also, different reference gene sets are used in 5E,F,G. Was the same approach of obtaining genes anti-correlated with CBS used in the in vivo studies?

-	Moreover, did the authors check the methylation in the gastric epithelia of the CBS deficient mice. This should be done to obtain the in vivo impact of CBS loss on methylation differences.

-	The paper stresses on two key impacts of CBS loss, one on methylation and the other on inflammation. Are these mechanisms independent or connected through inflammation? Increased inflammation is also associated with DNA methylation changes.

-	CBS loss, its alterations in homocysteine metabolism, and its impact on DNA methylation. To validate this, are the HC, methionine, SAM levels increased upon CBS KO in the cell line model? Can treating WT cells with HC phenocopy CBS loss?

-	The authors have identified a significant negative correlation between methylation and expression in BLCA, EAC, HNSC, LIHC, UCEC in comparing CIMP vs Non-CIMP samples (Fig. 3 A,B). However in case of HNSC, the baseline normals have reduced expression even though the methylation level is minimum compared to the CIMP and non-CIMP samples. It is better to also show these relations as correlation plots of methylation vs. expression.

-	Also, for HNSC, LIHC, THYM, and UCEC, the average methylation levels are around or below 0.2 for all samples, leading to a methylation difference of ~0.1 between CIMP and non-CIMP samples. Could the authors comment on the significance of the methylation effects at such low levels.

-	It is not obvious if the authors have analyzed glioblastoma and colorectal cancer for the CBS epimutations. Given the high CIMP content in these cancer types, one would expect to see a similar trend as in stomach and esophageal adenocarcinoma, if the authors want to generalize the role of CBS epimutations in association with CIMP in carcinogenesis.

**Reviewer 3**

**Are you able to assess all statistics in the manuscript, including the appropriateness of statistical tests used?** Yes, and I have assessed the statistics in my report.

**Comments to author:**

The work of Padmanabhan et al, described in Highly Recurrent CBS Epimutations Shape CpG Island Methylator Phenotypes and Inflammation in Gastric Cancer implicates the cystathionine beta-synthase gene in aberrant DNA methylation and inflammation in gastric cancer.

Summary: The authors examine the DNA methylation patterns and gene expression patterns in gastric cancer cell lines with CIMP or not, and controls. They demonstrate the CBS is hypermethylated in CIMP cell lines, with reduced gene expression and protein expression, compared to nonCIMP and normal cell lines. This loss of expression was dependent on methylation and disappeared when cells were treated with 5-azacytidine. Methylation was enriched at CpG islands compared to other locations in the genome. The CBS gene was included in a set of 18 genes identified from primary gastric cancers with decreased expression and hypermethylation, which also occurred in TCGA data. Diminished CBS staining was observed n microarrays compared to normal gastric epithelium. Hypermethylation and reduced expression were also identified in 5 other cancer type from TCGA data. Gastric intestinal metaplasia, a precursor condition to GC shared DNA methylation sites with GC including CBS. CBS was deleted in two cell lines for two clones using CRIPSR and assessed at multiple passages. Genomic methylation was altered, enriched at CGIs, and accompanied by metabolite disturbances. The loss of CBS activity correlated with reduced H2S, which could be corrected with administration of slow release H2S donors and lowered the elevated NFKB levels indicative of inflammatory response. Four other TCGA cancer types also showed this inverse relationship between inflammatory response and reduced CBS expression, as did the CCLE cell lines. Knockout of CBS in mice yielded RNA data from stomach that indicated roles in inflammatory pathways.

Comments: The authors identify a putative role for CBS in DNA methylation in gastric cancer and show data to indicate that it could also play a role in inflammatory processes. These characteristics would place the methylation-based repression of CBS as a functional contributor to epigenetic and inflammatory responses in gastric cancer, which has not been previously reported. The challenge faced by the authors is distinguishing correlated events from causal events. Most of the findings of the paper are correlative and not fully convincing of causal connections.

1. The foremost question is why the authors looked for a gene with promoter methylation as a likely cause of CIMP. If methylation is invoked, then some event causing aberrant methylation has to target the CBS promoter. In other examples of such events, promoter methylation accompanies gene mutation, as an alternative mechanism of eliminating gene expression, either of which is relevant to the condition. Here, the CBS gene mutations are deemed secondary to MSI.

2. The second question that arises from this work is why the authors invoke pan-cancer in the introduction when the data they provide indicates that no more than 5 cancer types show a correlation between hypermethylation and gene repression.

3. The definition of CIMP is quite variable in the literature. However, the authors have not established an objective measure of CIMP. The argument is that enrichment at CGIs implicates CIMP. However, many cancers have aberrant DNA methylation, but do not show CIMP. Their aberrant methylation largely can occur at CGIs, which are PRC2 targets. They may also some loss of methylation in open sea regions. For these reasons, the authors need to more clearly delineate what sites represent CIMP or non-CIMP conditions, and show quantitatively that methylation is conserved at these sites.

4. Many genes are correlated with altered methylation. Many cancer driver genes demonstrate a correlation with DNA methylation in at least one cancer type. To claim causality, the authors should show a direct relationship between DNA methylation and loss of function. Despite the compelling nature of the gene function, it is possible that the role of the gene has an impact on histone methylation, which indirectly affects DNA methylation, rather than a direct effect on DNA methylation. This illustrates how correlations can be vexing.

a. H3K9me3 also is a repressive mark, but only h3K27me3 was reported in the CIMP cell lines.

b. The literature referring to homocysteine accumulation, Choumenkovitch et al. indicates lowered genomic methylation in the homozygous KO mutant mice compared to WT mice, whereas here the loss of CBS leads to increased DNA methylation compared to non-CIMP samples, which seems to contradict the reference.

5. Significance after multiple test correction should be reported both in the text and in the tables, not one or the other location, for clarity. For example, data for Fig 5E,F, and G.

There are some technical issues with the text:

1. For example- In Fig S1. the branches of the dendrogram indicate four groups not three, depending on where the branches are cut, but it is not correct to say there are three groups.

2. Fig S1 should have labels to indicate MSI, EBV, known driver genes, etc. to be comprehensive.

3. Does Fig S1 show the 10,000 most variable sites, it appears to be fewer, the number should be reported.

4. In Fig 4F legend, what does "annotated" mean?

5. In figure 2A what data is being compared for significance values?

6. Figure S3 has no positive control for H3K27me3.

7. Why aren't the methylation data for samples in Fig 1C shown along with data for Fig 1D?

8. Page 3 should not use the word "personified" to describe CIMP.

9. The clinical persistence of tumors with CBS hypermethylation is only a correlation and does not indicate a functional role for CBS in malignancy risk. Multi-variate survival curves would be necessary to make that claim. It may simply be a phenotype of the subtype of tumors.

10. Fig 4E- these samples are derived from timepoints of the same clones and should not be presented as independent data.

**Authors Response**

**Point-by-point responses to the reviewers’ comments:**

**Reviewer #1:**

*This study clearly demonstrated a novel epigenetic alteration which could potentially lead to development of CpG island methylator phenotype-positive gastric cancer. This study is well designed, experiments were adequately performed, and results are solid to support the conclusion. I agree to accept this manuscript.*

Response: We thank the Reviewer for the positive feedback and acceptance of the manuscript.

**Reviewer #2:**

*The authors associate loss of CBS, by epigenetic silencing (epimutation), to the CIMP phenotype in gastric cancers and other cancers. Deleting CBS by CRISPR causes increased methylation resembling CIMP. Mechanistically, the authors argue that CBS loss alters methionine and homocysteine metabolism, which may cause the methylation changes. The authors show that CBS transcriptional loss as the most frequent and specific marker 50 GC CIMP-high cell lines from a panel of 50 GC cell lines. Using priary GC cancer datasets from TCGA and Singapore cohort, they show that CBS methylation/silencing associated with CIMP phenotype. About 1/3rd of all cancers are observed to have CBS downregulation in their CIMP+ counterparts. Hypermethylated CGIs in CBS deficient cells have a good 38% overlap with the commonly hypermethylated CGIs in patient GIM and GC samples. Overall the manuscript is well presented. Mechanisms underlying CIMP are of general interest to the field. The proposed role of CBS in causing CIMP is a novel observation and deserves careful consideration and publication. Following are some concerns that need to be addressed.*

Response: We thank the Reviewer for the valuable feedback. We hope that our revisions will be sufficient to address the concerns, especially given the challenges and limitations imposed by COVID. While we have made best attempts to perform new experiments requested by the Reviewer, our efforts have been significantly constrained by the COVID-19 crisis, leading to extremely stringent national and institutional restrictions on laboratory access, retrieval of material, and available staff (many of whom needed to be rechanneled to COVID-19 activities or were subjected to stay-at-home lockdown). Nonetheless, we have added new experimental components in our revised manuscript, including preliminary DNA methylation data in *CBS*-deficient animal tissue and metabolic analysis in *CBS*-deficient cell lines.

- *Mechanistic link to activation of inflammatory pathway by reduction in H2S levels is not clear.*

Response: We have addressed this point in results (Pg12), “H_2_S is a gasotransmitter, and the CBS-H_2_S axis has been implicated in multiple physiological processes including inflammation, vasodilation, and cell metabolism, in part through H_2_S’s function of ‘sulfhydration’ - a post-translational modification on proteins [1-3]”. The discussion (Pg16) further expands on this point - “Our findings in *CBS*-deficient gastric cells, that lower H_2_S levels are associated with increased NF-kB activity, draws from multiple studies in other cell line and animal model systems that have investigated this mechanistically ([4-7]). These previous studies have shown the anti-inflammatory function of *CBS* loss and H_2_S is multifaceted, involving the following: blocking NF-kB p65 activation, regulating cellular redox status, DNA damage, stabilization of hypoxia-inducible factor (HIF)-1α, regulating immune cell migration, mitochondrial electron transport and apoptosis. At the molecular level, H_2_S’s role in ‘sulfhydration’, has been linked to some of these biological effects [8]. Future work in the *CBS*-deficient *in vitro* model will systematically investigate the role of these molecular mechanisms in inducing inflammatory responses.”

*Also, different reference gene sets are used in 5E,F,G. In the in vivo experiments with CbS-/- mice, the inflammatory pathways identified are not the same as that identified in cell lines data sets (5E, F). Thus, the impact of CBS on activating inflammatory response, should be carefully interpreted/stated in the paper, with the proper caveats to this interpretation given that the data is not conclusive.*

Response: Thank you for this comment. The *in vivo* experiments with *CBS*-deficient mice samples in Figure 5G were analysed using the PANTHER annotation tool, whereas the data shown in Figure 5A, E, F relating to human samples were analysed using MSigDB hallmark gene set (curated human gene list). In the revised manuscript, we have re-analysed the *CBS*-deficient mice gene expression data in Figure 5G using the mouse-human gene ID conversion tool and MSigDB hallmark gene sets, to ensure a like-to-like comparison. We found similar results in this new analysis, which indicated ‘*Inflammatory response*’ and ‘*TNFA signalling via NF-κB*’ pathways as significantly upregulated (q-value <0.01), consistent with the cell line data sets (Figure 5A,E,F). The new data is included in Supplementary Table 22, with manuscript methods (Pg.22) and results (Pg14) updated, “In addition, pathway analysis using MSigDB hallmark gene sets also indicated ‘*Inflammatory response*’ and ‘*TNFA signalling via NF-κB*’ as significantly upregulated (q-value <0.01) (Supplementary Table 22)”. As suggested by the Reviewer, we have also carefully re-phrased our interpretation of the inflammation-related findings in the abstract (Pg2), “In a murine genetic model of *CBS*-deficiency, preliminary data indicated upregulated immune-mediated transcriptional signatures in the stomach” and also discussion (Pg16), “Preliminary investigations in *CBS*-deficient mouse stomachs indicated upregulation of several immune-related processes.”

*Was the same approach of obtaining genes anti-correlated with CBS used in the in vivo studies?*

Response: Expression-correlation analysis is considered a robust way to gauge functional associations in large datasets [9], and so we utilised this approach for all primary tumour samples, and CCLE cell lines based on the “co-expression function” data on cbioportal (Spearman correlation, q<0.05). In contrast, a fold-difference method was used for the genetic mouse model and *CBS-*deficient cell line datasets (wherein *CBS* was experimentally modulated to create *CBS*-proficient and *CBS*-deficient groups) as these involved smaller sample sizes that precluded reliable correlation analysis and multiple testing [10].

- *Moreover, did the authors check the methylation in the gastric epithelia of the CBS deficient mice. This should be done to obtain the in vivo impact of CBS loss on methylation differences.*

Response: The Tg-hCBS Cbs^−/−^ mouse model presented in this manuscript was previously derived in Prof. Kruger’s Laboratory [11]. Cbs^−/−^ mice, originally created by Maeda [12], exhibit a high degree of neonatal lethality. To rescue this lethality, Kruger’s group generated Tg-hCBS transgenic mouse carrying a human CBS cDNA construct under a zinc-inducible metallothionein promoter, and crossed them to Cbs^−/−^ mice, to generate heterozygous Tg-hCBS Cbs^+/−^ mice. Tg-hCBS Cbs^+/−^ mice were then intercrossed on zinc water to produce Tg-hCBS Cbs^−/−^ mice that were born in a normal Mendelian ratio and survived into adulthood. Adult phenotypes of *CBS*-deficiency were studied by discontinuing zinc supplementation after Tg-hCBS Cbs^−/−^ mice were weaned (~1 month old). As expected, Tg-hCBS Cbs^−/−^ adults develop severe hyperhomocysteinemia [11].

In this manuscript, we presented H&E data pertaining to 11 genotyped Tg-hCBS Cbs^−/−^ stomachs (derived from Tg-hCBS Cbs^−/−^ intercrosses), and RNA-sequencing data pertaining to 2/11 Tg-hCBS Cbs^−/−^ stomachs using whole FFPE tissue sections. In order to select samples for methylation analysis, we first attempted to validate loss of mouse *Cbs* protein using a Rabbit polyclonal antibody (Protein tech, 14787-1-AP). Unfortunately, optimisation of this antibody for immunohistochemistry was not successful. We could not use the CBS antibody utilized for our human cell line work since it was a mouse anti-human CBS antibody (Abnova, H00000875-M01). Due to limited time and non-availability of mouse fresh/frozen tissue samples in the COVID period, we proceeded to select 4 Cbs^+/+^ and 6 Cbs^-/-^ FFPE samples (> 4 months of age, >3 months of zinc-water cessation, *Table A*) and performed methylation profiling using DNA from whole FFPE sections on the newly launched Illumina mouse methylation array platform (Illumina, 20041558). The array contains 286,916 CpG probes (0.3X of the Infinium human methylation array) spread across different features such as coding and non-coding TSS, imprinted genes, etc. Differential methylation analysis between Cbs^+/+^ and Cbs^-/-^ groups (β-value difference ≥ 0.1 and q-value ≤0.05) indicated 1053 CpG sites altered in Cbs^-/-^samples, of which 165 CpG sites were hypermethylated (15.6%) and 888 CpG sites were hypomethylated (84.3%). Analysis of the differentially methylated CpGs based on CpG context (binomial test, two-sided) indicated significant depletion of hypomethylated CpG sites in CpG islands (P = 1.73×10^-12^), and enrichment of hypomethylated CpG sites at CpG shelf (P = 9.5×10^-8^), and open sea regions (P = 0.005) (*Figure A*). However, no significant enrichment or depletion was seen for hypermethylated CpG sites in Cbs^-/-^ samples, unlike our findings from our human *in vitro* model.

Although Cbs^-/-^ tissues exhibit DNA methylation changes, we are cautious in interpreting these findings. First, the efficiency of mouse *Cbs* knockdown in samples used for the methylation analysis has not been tested, as opposed to the complete loss of *CBS* protein in the human cell line model. Moreover, residual activity of the human CBS transgene (i.e. leaky expression in the absence of zinc supplementation) in the Tg-hCBS Cbs^−/−^ mouse model has been observed in a proportion of mouse livers but has not yet been characterized in stomach tissue. Second, the methylation signal in the mouse experiment represents whole FFPE sections (i.e., with differing cellularity of epithelial and other cell types) and it is possible that the signal from gastric epithelial cells is diluted. Third, whether the trends in methylation data are truly representative of the mouse model is unclear, and will require higher sample numbers that particularly account for older age and gender [13]. For example, a recent study by Kruger’s group in the Tg-hCBS Cbs^−/−^  zinc-inducible model of *CBS*-deficiency for only two weeks, reported predominant hypermethylation (89% of total change) at a subset of promoter CpG sites using a MeDIP-array platform, but no changes in DNA methylation ‘levels’ in DNA extracted from frozen liver tissue using LC-MS/MS, [14], highlighting the differences in study approaches and results. Given these caveats, we believe that the Tg-hCBS Cbs^−/−^ stomach mouse methylation data is preliminary and have presented this data to the Reviewer in an open and transparent manner but have not included it in the revised manuscript. We believe that a more systematic study, that also includes other *CBS*-mutant models [15], is needed to address the overall impact of *CBS* deficiency in mice.

To account for these pilot findings, we have carefully re-phrased our entire manuscript to highlight that *CBS* deficiency in human gastric cells results in abnormal DNA methylation patterns *in vitro*. We have also modified our discussion (Pg14), “*In vitro* loss of CBS in normal human gastric epithelial cells is associated with both aberrant DNA methylation and an inflammatory response, indicating a novel bi-faceted function to this epimutation. Whether these *in vitro* phenotypes are observed in mouse models, and/or exacerbate with prolonged periods of time, following a ‘methylation accumulation model’ needs to be ascertained, given that primary lesions also take years to develop.”

***Table A*.** List of samples used for the methylation analysis.

| ***Cbs*^+/+^ n=4**  **(Gender, age)** | **Cbs^−/−^ n=6**  **(Gender, age)** |
| --- | --- |
| Female, 8.1 months | Female, 11.1 months |
| Female, 8.1 months | Female, 11.1 months |
| Female, 8.1 months | Female, 11.1 months |
| Male, 4.4 months | Male, 7.5 months |
|  | Male, 5.7 months |
|  | Male, 5.7 months |

***Figure A***. Distribution of differentially methylated CpG sites in Cbs^−/−^ samples. ‘Annotated’ refers to the distribution of CpG probes on the mouse methylation array. P<0.05.

- *The paper stresses on two key impacts of CBS loss, one on methylation and the other on inflammation. Are these mechanisms independent or connected through inflammation? Increased inflammation is also associated with DNA methylation changes.*

Response: Thank you for this comment. We entirely agree that inflammatory processes may account for a portion of the observed DNA methylation changes in *CBS*-deficient cells, potentially linking the phenotypes. We have re-phrased our discussion and included specific references that allude to this point (Pg15), “While our study has not identified the exact mechanism by which *CBS*-deficiency results in abnormal DNA methylation, we speculate that the phenomenon may be complex, involving metabolic perturbations in the transmethylation cycle (e.gs, other classes of methyl group acceptors such as histones, RNA, lipids), and perhaps also the transsulfuration pathway (e.gs H_2_S, glutathione, inflammatory changes) since several of these components have been previously identified as modifiers of DNA methylation, through direct and indirect mechanisms [16-21].”

- *CBS loss, its alterations in homocysteine metabolism, and its impact on DNA methylation. To validate this, are the HC, methionine, SAM levels increased upon CBS KO in the cell line model?*

Response: Thank you for this comment. In the revised manuscript, we have now performed targeted metabolic assays to measure homocysteine, SAM, SAH and methionine in the GES1 cell line model. The methods (Pgs.27-31) and results (Pg11) are updated (revised Figure 4H), “Since excess homocysteine is usually exported out of the cell, we then measured homocysteine, SAM, S-adenosyl homocysteine [SAH] and methionine in culture media from the GES1 cell line model at 24 hours using targeted assays (Methods). We found significant increases in homocysteine (FD 1.28; P<0.05) and SAM (FD 1.50; P<0.05), but not SAH and methionine in *CBS*-deficient cells (Figure 4H, middle panel), further suggestive of an imbalance in the methyl group cycle.” Thus, these observations further support the idea that *CBS*-deficient cells have altered methyl group homeostasis. As mentioned in the discussion, the concentration of methionine in culture media is very high and may mask underlying changes (Pg15), “For example, it will be useful to assess metabolic changes in *CBS*-deficient cells grown in media with lower methionine levels, since normal media is considered to contain supraphysiological levels of methionine and could thus blunt the severity of phenotypes assessed [22]”.

- *Can treating WT cells with HC phenocopy CBS loss?*

Response: We agree with the Reviewer that this is an interesting question to answer. However, to effectively address this point, we would need to first generate and characterize a homocysteine model with different doses in normal gastric cells, since conflicting effects on cell proliferation have been reported [23-25]. We would then have to perform genome-wide methylation profiling and inflammation-related assays at various passage numbers, similar to the experimental design utilized for the *CBS*-deficient model. This would involve extensive experimental work. We acknowledge the relevance of this experiment, and refer to it in discussion (Pg15), “Further functional studies that mimic the metabolic states associated with *CBS*-deficiency (e.g. homocysteine exposure) and culture manipulations are required to identify the precise metabolic sequelae leading to altered methylation”.

- *The authors have identified a significant negative correlation between methylation and expression in BLCA, EAC, HNSC, LIHC, UCEC in comparing CIMP vs Non-CIMP samples (Fig. 3 A,B). However in case of HNSC, the baseline normals have reduced expression even though the methylation level is minimum compared to the CIMP and non-CIMP samples. It is better to also show these relations as correlation plots of methylation vs. expression.*

Response: We agree with the Reviewer and have now included correlation plots for BLCA, EAC, HNSC, LIHC, UCEC and STAD as a new Supplementary Figure 5.

- *Also, for HNSC, LIHC, THYM, and UCEC, the average methylation levels are around or below 0.2 for all samples, leading to a methylation difference of ~0.1 between CIMP and non-CIMP samples. Could the authors comment on the significance of the methylation effects at such low levels.*

Response: Thank you for this comment. Our analysis of *CBS* promoter methylation levels between CIMP and non-CIMP categories (Wilcoxon rank-sum test, P<0.05) revealed significant hypermethylation only in BLCA, EAC, HNSC, LIHC and UCEC, but mild hypomethylation in THYM (Pg7, Figure 3B). Since average methylation levels of a group do not reflect the spread of individual values, we have now represented the data as a percentage of cases in CIMP and non-CIMP categories with *CBS* promoter methylation levels in a defined β-value range (0-0.2, 0.2-0.4, 0.4-0.6, 0.6-0.8, 0.8-1.0) (new Supplementary Figure 4). For example, in STAD, 98.8% of CIMP tumours have *CBS* promoter β-value of > 0.2, compared to 49.2% of non-CIMP tumours. Although the percentage varies for each tumour type,

“CIMP tumours tend to carry higher *CBS* promoter β-values vs. non-CIMP tumours (BLCA: 63.8% vs. 17.1%, UCEC: 29.3% vs. 1.80%, EAC: 93.0% vs. 37.90%, HNSC: 76.7% vs. 17.7%, LIHC: 28.8% vs. 15.6%, STAD: 98.8% vs. 49.2%)” (Supplementary Figure 4 legend) (Pg48).

- *It is not obvious if the authors have analyzed glioblastoma and colorectal cancer for the CBS epimutations. Given the high CIMP content in these cancer types, one would expect to see a similar trend as in stomach and esophageal adenocarcinoma, if the authors want to generalize the role of CBS epimutations in association with CIMP in carcinogenesis.*

Response: Thank you for this comment. To clarify the list of cancers included for the pan-cancer analysis, we have updated the Methods section (Pgs.21-22), “For the pan-cancer analysis, 22 cancers (TCGA abbreviations: LIHC, HNSC, ESCA, THYM, BLCA, UCEC, ACC, LGG, BRCA, CESC, CHOL, COAD-READ, GBM, KIRP, LUAD, MESO, PAAD, PRAD, SARC, SKCM, TGCT, and UVM) were analysed for differential gene expression analysis of *CBS* in most ‘hypermethylated/CIMP’ vs. other methylation categories (Wilcoxon rank-sum test, P<0.05), as defined by TCGA (The Cancer Genome Atlas) or by Karpinski et al (for UCEC) [26] via MEXPRESS[27]. For UCEC, the TCGA-CIMP sample labels were not publicly available, and therefore, we used CIMP sample labels from Karpinski et al (a pan-cancer study of CIMP)”.

In the initial differential gene expression analysis using TCGA-CIMP labels, no significant changes were seen in COAD-READ (*Figure A*, Upper Panel). However, since COAD-READ CIMP displayed a trend for lower *CBS* levels, we repeated the differential gene expression analysis using CIMP-labels from Karpinski et al. and found significant downregulation of *CBS* (P = 0.02) (*Figure A*, Lower panel). This discrepancy may be due to differences in CIMP categorisation by earlier studies (i.e. while TCGA demarcated CIMP-High [CIMP], and non-CIMP [CIMP-Low, Cluster 3 and Cluster 4], Karpinski et al, provided a single CIMP grouping). Interestingly, *CBS* promoter methylation was significantly higher in COAD-READ CIMP (P = 4.8×10^-21^) (*Figure B*). Correlations between methylation and gene expression showed a trend for inverse association (r = -0.09, P = 0.06) (*Figure C*), which we verified using TCGA (Firehouse legacy) data from cbioportal (n=372 patients, r = -0.52, P = 2.71 ×10^-27^) (*Figure D*).

For GBM and LGG, the initial differential gene expression analysis using TCGA-CIMP labels indicated no significance change in GBM, but higher *CBS* levels in LGG CIMP (*Figure A*, Upper panel). Again, *CBS* promoter methylation was significantly higher in CIMP categories of both tumour types compared to its non-CIMP counterpart (GBM [P = 0.0006], LGG [P = 5.9×10^-32^]) (*Figure B*). Correlation between methylation and gene expression did not show a significant inverse association in GBM (r = -0.10, P = 0.4) and LGG (r = 0.12, P = 0.03) (*Figure C*), whereas correlations using TCGA (Firehouse legacy) data indicated significant inverse correlation in LGG (n= 516 patients, r = -0.36, P = 6.08 ×10^-18^), but not in GBM (n=136 patients, r = -0.19, P = 0.1) (*Figure D*).

While observations in COAD-READ were overall consistent, findings from GBM/LGG may be confounded by sample size, tumour categorisations and pooled CBS gene expression signal from different cell types in the tumour microenvironment [28, 29]. Nevertheless, it was interesting to note that amongst the three tumour types, COAD-READ, a gastrointestinal cancer showed similar trends to STAD. Future work involving in-depth characterisation of primary data and cell line models may help to shed light on the role of *CBS* epimutations and CIMP in these tumour types.

***Figure****.* Gene expression and methylation status of *CBS* in COAD-READ, GBM and LGG.

**Reviewer #3:**

*The work of Padmanabhan et al, described in Highly Recurrent CBS Epimutations Shape CpG Island Methylator Phenotypes and Inflammation in Gastric Cancer implicates the cystathionine beta-synthase gene in aberrant DNA methylation and inflammation in gastric cancer.*

*Summary: The authors examine the DNA methylation patterns and gene expression patterns in gastric cancer cell lines with CIMP or not, and controls. They demonstrate the CBS is hypermethylated in CIMP cell lines, with reduced gene expression and protein expression, compared to nonCIMP and normal cell lines. This loss of expression was dependent on methylation and disappeared when cells were treated with 5-azacytidine. Methylation was enriched at CpG islands compared to other locations in the genome. The CBS gene was included in a set of 18 genes identified from primary gastric cancers with decreased expression and hypermethylation, which also occurred in TCGA data. Diminished CBS staining was observed n microarrays compared to normal gastric epithelium. Hypermethylation and reduced expression were also identified in 5 other cancer type from TCGA data. Gastric intestinal metaplasia, a precursor condition to GC shared DNA methylation sites with GC including CBS. CBS was deleted in two cell lines for two clones using CRIPSR and assessed at multiple passages. Genomic methylation was altered, enriched at CGIs, and accompanied by metabolite disturbances. The loss of CBS activity correlated with reduced H2S, which could be corrected with administration of slow release H2S donors and lowered the elevated NFKB levels indicative of inflammatory response. Four other TCGA cancer types also showed this inverse relationship between inflammatory response and reduced CBS expression, as did the CCLE cell lines. Knockout of CBS in mice yielded RNA data from stomach that indicated roles in inflammatory pathways.*

*Comments: The authors identify a putative role for CBS in DNA methylation in gastric cancer and show data to indicate that it could also play a role in inflammatory processes. These characteristics would place the methylation-based repression of CBS as a functional contributor to epigenetic and inflammatory responses in gastric cancer, which has not been previously reported. The challenge faced by the authors is distinguishing correlated events from causal events. Most of the findings of the paper are correlative and not fully convincing of causal connections.*

Response: We thank the Reviewer for the valuable feedback. As suggested by the Reviewer, we have re-phrased the text in the entire manuscript to emphasize findings as being more ‘associative’ than ‘causal’ in appropriate contexts. However, we would also like to highlight our findings from the *CBS* CRISPR cell line model which do support a causative role for *CBS* in the observed phenotypes. We hope that our revisions will be sufficient to address the concerns, especially given the challenges and limitations imposed by COVID, such as stringent national and institutional restrictions on laboratory access, retrieval of material, and available staff (many of whom needed to be rechanneled to COVID-19 activities or were subjected to stay-at-home lockdown). Nonetheless, we have included new experimental data in our revised manuscript involving targeted metabolic analysis in the *CBS*-deficient cell line model.

1. *The foremost question is why the authors looked for a gene with promoter methylation as a likely cause of CIMP.*

Response: Thank you for this comment. Our rationale to investigate ‘epimutations’ as contributors to the CIMP phenotype in GC stemmed from two major lines of reasoning: (1) Prior large-scale studies (e.gs. gastric TCGA [30] and pan-cancer [31]) have not identified a genetic driver of CIMP in GC (2). In the absence of a genetic event, we chose to focus on ‘epimutations’, particularly driven by DNA hypermethylation, since it is a well-established and active mechanism of gene silencing, as exemplified by *MLH1* promoter hypermethylation which is responsible for the MSI molecular phenotype in GC [32].

*If methylation is invoked, then some event causing aberrant methylation has to target the CBS promoter.*

Response: We agree that the upstream molecular processes driving aberrant *CBS* promoter hypermethylation is still unknown. Having identified the importance of *CBS* epimutation in this manuscript, we aim to investigate the molecular mechanisms governing *CBS* gene regulation in a future study. We speculate on this point in discussion (Pg14), “The tissue-independent epigenetic targeting of *CBS* may be triggered either by a fundamental cellular insult, dysregulation of factors impacting *CBS* expression including non-coding RNAs[33], or perhaps as an adaptation to disturbances in biochemically connected pathways such as methionine levels[34, 35]”. It is entirely possible that this upstream event may be linked or independent to the methylation changes resulting from *CBS* epimutation. Nevertheless, irrespective of the nature of the causative event, we would like to emphasize that the consequences of *CBS* deficiency are of relevance, given that the CIMP tumour phenotype is based on an accrual model of abnormal DNA methylation observed in GC pre-malignant conditions such as intestinal metaplasia.

*In other examples of such events, promoter methylation accompanies gene mutation, as an alternative mechanism of eliminating gene expression, either of which is relevant to the condition. Here, the CBS gene mutations are deemed secondary to MSI*.

Response: Thank you for this comment. While we concur that some genes may be inactivated either by promoter methylation [32] or genetic mutation (e.g. *CDH1* (7.9%) [36] (*Figure A*, left panels), or by both, this may not be a generalised phenomenon in GC. For examples, *MLH1* (1.5%) [37], *MGMT* (0.9%) [38] and *CDKN1C* (0.3%) are predominantly inactivated by promoter methylation [39] as evidenced by the low frequency of genetic mutation (*Figure A*, left panels). Moreover, the association of these mutations with MSI distinctly varies in GC (*Figure A*, right panels), with *MLH1* mutations displaying a tight association with this subtype. Similar to these examples, *CBS* gene mutations (2.4%) represent a minor mechanism of gene silencing compared to the frequency of promoter hypermethylation (Pgs.6-7). While *CBS* somatic mutations significantly associate with MSI cases, the lack of microsatellite repeat regions in the coding regions of the gene (*Figure B*), make it unlikely that the observed CBS mutations are an indirect consequence of MSI. Moreover, our observation of *CBS* *germline* mutations also precludes MSI being a causative process, as the latter is a somatic event. Since we do not know for certain if *CBS* somatic mutations precede or are subsequent to the onset of MSI, we have modified the text in the manuscript to reflect either scenario (Pg7), i.e. “Mutated cases were heterozygous, with a low prevalence (6/243, 2.46% frequency), co-occurred with gastric CIMP and MSI (Fisher’s exact test, P = 0.001), and exhibited promoter hypermethylation (β-value range: 0.59 - 0.79), suggesting that genetic mutations are much less frequent than epimutations at the *CBS* locus and may or may not represent passenger events owing to MSI.”

***Figure A.*** Occurrence of mutation and methylation in select genes in gastric TCGA from cbioportal. Data in left panels were derived from Firehouse legacy (n=328). Data in right panels were derived from gastric TCGA study (n=295) [30].

***Figure B.*** UCSC Genome Browser track of CBS (GRCh38/hg38) with microsatellites represented.

2. *The second question that arises from this work is why the authors invoke pan-cancer in the introduction when the data they provide indicates that no more than 5 cancer types show a correlation between hypermethylation and gene repression.*

Response: We agree with the Reviewer and have replaced the words ‘pan-cancer role’ with ‘broader role’ (Pgs.4,14).

3. *The definition of CIMP is quite variable in the literature. However, the authors have not established an objective measure of CIMP. The argument is that enrichment at CGIs implicates CIMP. However, many cancers have aberrant DNA methylation, but do not show CIMP. Their aberrant methylation largely can occur at CGIs, which are PRC2 targets. They may also some loss of methylation in open sea regions. For these reasons, the authors need to more clearly delineate what sites represent CIMP or non-CIMP conditions, and show quantitatively that methylation is conserved at these sites.*

Response: Thank you for this comment. We agree with the Reviewer that the definition of CIMP has evolved over the years, with variability observed in earlier studies that used small gene-specific panels to a more consistent categorisation of tumours after the advent of genome-wide methylation arrays [40]. In GC, the most comprehensive study in primary tumours till date is the TCGA, that has used genome-wide methylation data to identify at least two major CIMP groups, i.e. EBV-CIMP and gastric-CIMP, supporting earlier studies [30, 41, 42]. Therefore, we used the gastric TCGA study as an objective measure of CIMP patterns and showed a significant overlap between GC CIMP signatures and *CBS*-deficient hypermethylation in CGIs (~38%, P<1×10^-4^) (Pg10) (Figure 4G). We also acknowledge that non-CIMP GC tumours harbour abnormal DNA methylation, but the genome-wide frequency and pattern is distinctly different from CIMP tumours. We verified that hypermethylated signatures in primary TCGA and gastric IM CIMP samples are significantly enriched for CGIs but depleted for Open Sea regions (compared to non-CIMP, Figure 3C). The pan-cancer analysis was also performed using TCGA-defined methylation categories (Pgs.21-22) that were identified using genome-wide methylation arrays.

4. *Many genes are correlated with altered methylation. Many cancer driver genes demonstrate a correlation with DNA methylation in at least one cancer type. To claim causality, the authors should show a direct relationship between DNA methylation and loss of function. Despite the compelling nature of the gene function, it is possible that the role of the gene has an impact on histone methylation, which indirectly affects DNA methylation, rather than a direct effect on DNA methylation. This illustrates how correlations can be vexing*.

Response: Thank you for this comment. We agree that several genes, including cancer drivers, are correlated with altered methylation [32] . However, our discovery analysis using three omics platform (i.e., DNA methylation, RNA expression and Proteomics) in the GC *in vitro* model identified *CBS* as the most recurrent loss-of-function epimutation associated with the CIMP class. Furthermore, we would like to point out that findings from the *CBS* CRISPR cell line model support a causative role for *CBS* in the observed phenotypes.

We also agree that *CBS* loss can impact histone methylation, which in turn could have an indirect effect on DNA methylation. We have re-phrased the discussion to specifically reflect this perspective (Pg15), “While our study has not identified the exact mechanism by which *CBS*-deficiency results in abnormal DNA methylation, we speculate that the phenomenon may be complex, involving metabolic perturbations in the transmethylation cycle (e.gs, other classes of methyl group acceptors such as histones, RNA, lipids), and perhaps also the transsulfuration pathway (e.gs H_2_S, glutathione, inflammatory changes) since several of these components have been previously identified as modifiers of DNA methylation, through direct and indirect mechanisms [16-21]”). Addressing causality between different epigenetic modifications has proved challenging even for specific loci. Thus, profiling multiple histone marks across passages in *CBS*-deficient cells would not only involve extensive work but would primarily serve to indicate genome-wide correlations rather than causality. Since SAM is a universal methyl group donor, a change in methyl group homeostasis is likely to affect different classes of downstream methyl group acceptors (e.gs, RNA [43-45], histones [46-48], lipids [49-51]) that may exert effects on the epigenome. For example, we have shown increased levels of 1-methyl nicotinamide (methyl group acceptor is nicotinamide) in *CBS*-deficient cells, a metabolite that has been shown to cause DNA hypermethylation in a different cell line [52]. This hypothesis is also supported by new experimental data (Pg11) (revised Figure 4H), which shows significant increases in key metabolites, homocysteine (FD 1.28; P<0.05) and SAM (FD 1.50; P<0.05) in GES1 *CBS*-deficient samples, further suggesting that *CBS* disruption results in an imbalance in the methyl group cycle.

a. *H3K9me3 also is a repressive mark, but only h3K27me3 was reported in the CIMP cell lines.*

Response: Our rationale for assessing the repressive H3K27me3 modification was based on prior data that implicated PRC2 (the complex that catalyses this histone mark) targets in CIMP tumours, (Pgs.5-6), “Since GC CIMP signatures are typically enriched for PRC2 targets which predominantly trimethylate histone H3 on lysine 27 [42, 53], we assessed H3K27me3 status at the *CBS* gene promoter in 2 gastric CIMP cell lines”. We agree that H3K9me3 may be altered at the *CBS* promoter and we have re-written the text to include this possibility (Pg6), “ However, we found no enrichment of this histone mark (Supplementary Figure 3), reinforcing a stronger role for DNA methylation in *CBS* silencing, likely in concert with other repressive histone marks (e.g. H3K9me3).

b. *The literature referring to homocysteine accumulation, Choumenkovitch et al. indicates lowered genomic methylation in the homozygous KO mutant mice compared to WT mice, whereas here the loss of CBS leads to increased DNA methylation compared to non-CIMP samples, which seems to contradict the reference.*

Response: We cited Choumenkovitch et al. to show that *CBS*-deficiency can result in tissue-specific changes in DNA methylation levels. In this study, the authors investigate 3-9 month old *CBS*-deficient mice and reported increased plasma homocysteine levels and a significant decrease in 5mc ‘levels’ in liver (but not brain) as measured from a ‘methyl acceptance assay’. This assay is an overall readout of pre-existing methylation levels and does not indicate the altered distribution of the methyl groups within the genome [54]. A recent study in the Tg-hCBS Cbs^−/−^  zinc-inducible model of *CBS*-deficiency for only two weeks, reported no change in DNA methylation ‘levels’ in liver using LC-MS/MS, but significant hypermethylation at a subset of promoter CpG sites using a MeDIP-array platform [14], highlighting the differences in study approaches and results. Our investigation using EPIC methylation arrays in human gastric *CBS*-deficient cells across ~3 months resulted in conserved hyper- (9171 CpG sites in GES1, 3645 CpG sites in HFE145) and hypo-methylation (7919 CpG sites in GES1, 3241 CpG sites in HFE145) changes to a similar extent. Thus, it is possible that cell type, duration of loss, model system and species influence molecular responses to *CBS* deficiency. We have re-phrased our discussion to address this point and clarify that CBS loss is associated with aberrant DNA methylation (i.e. both hyper- and hypo) (Pg14), “*In vitro* loss of CBS in normal human gastric epithelial cells is associated with both aberrant DNA methylation and an inflammatory response, indicating a novel bi-faceted function to this epimutation. Whether these *in vitro* phenotypes are observed in mouse models, and/or exacerbate with prolonged periods of time, following a ‘methylation accumulation model’ needs to be ascertained, given that primary lesions also take years to develop”.

5. *Significance after multiple test correction should be reported both in the text and in the tables, not one or the other location, for clarity. For example, data for Fig 5E,F, and G.*

Response: We have updated the manuscript with significance after multiple testing correction in the text, figures and tables.

*There are some technical issues with the text:*

1. *For example- In Fig S1. the branches of the dendrogram indicate four groups not three, depending on where the branches are cut, but it is not correct to say there are three groups.*

Response: We have re-written the methods (Pg19) and modified Figure S1 and its legend (Pg45) to address this point.

2. *Fig S1 should have labels to indicate MSI, EBV, known driver genes, etc. to be comprehensive.*

Response: We have altered Figure S1 to include EBV, MSI and the mutational status of 3 cancer genes (*TP53*, *AR1D1A*, *PIK3CA*).

3. *Does Fig S1 show the 10,000 most variable sites, it appears to be fewer, the number should be reported.*

Response: Figure S1 represents the top 10,000 most variable sites as mentioned in the figure legend.

4. *In Fig 4F legend, what does "annotated" mean?*

Response: Please refer to the figure legend for Figure 4F (Pg42), ‘Annotated’ refers to the distribution of CpG probes on the Infinium methylation EPIC array’, i.e. it represents all the probes on the EPIC array platform.

5. *In figure 2A what data is being compared for significance values?*

Response: In Figure 2A (right panel), each CIMP group (i.e. EBV-CIMP and gastric-CIMP) is being compared to non-CIMP. Figure 2A and its legend (Pg40) has been revised for clarity.

6. *Figure S3 has no positive control for* *H3K27me3.*

Response: We have included positive controls for H3K27me3-ChIP (*ZRANB2-AS1, LHFPL4)* that were selected from a prior study [55]. Supplementary Figure 3, its legend (Pg47) and methods (Pg25) have been modified accordingly.

7. *Why aren't the methylation data for samples in Fig 1C shown along with data for Fig 1D?*

Response: We have added the methylation data for samples in Figure 1D and modified its figure legend (Pg39).

8. *Page 3 should not use the word "personified" to describe CIMP.*

Response: We have replaced “personified” with “characterized” on Pg3.

9. *The clinical persistence of tumors with CBS hypermethylation is only a correlation and does not indicate a functional role for CBS in malignancy risk. Multi-variate survival curves would be necessary to make that claim. It may simply be a phenotype of the subtype of tumors.*

Response: We acknowledge this point and have eliminated the sentence, ‘implying a potential role in malignancy risk’ in the results section on Pg8.

10. *Fig 4E- these samples are derived from timepoints of the same clones and should not be presented as independent data.*

Response: We have modified the labels in Figure 4E and its legend (Pg42) (“Unsupervised clustering of conserved hypermethylated 9171 CpG sites in GES1 *CBS*-deficient clones [Clone 1, C1; Clone 2, C2] across passages”). Since culturing time can be a significant variable in epigenetic studies, we have included the passage number to highlight that the hypermethylated signature is observed in all passage numbers of *CBS*-deficient clones and absent in controls.

**References**

1.	Paul BD, Snyder SH: **H2S signalling through protein sulfhydration and beyond.** *Nature Reviews Molecular Cell Biology* 2012, **13:**499.

2.	Bourque C, Zhang Y, Fu M, Racine M, Greasley A, Pei Y, Wu L, Wang R, Yang G: **H2S protects lipopolysaccharide-induced inflammation by blocking NFκB transactivation in endothelial cells.** *Toxicology and Applied Pharmacology* 2018, **338:**20-29.

3.	Robert K, Nehme J, Bourdon E, Pivert G, Friguet B, Delcayre C, Delabar JM, Janel N: **Cystathionine beta synthase deficiency promotes oxidative stress, fibrosis, and steatosis in mice liver.** *Gastroenterology* 2005, **128:**1405-1415.

4.	Singh SB, Lin HC: **Hydrogen Sulfide in Physiology and Diseases of the Digestive Tract.** *Microorganisms* 2015, **3:**866-889.

5.	**A Review of Hydrogen Sulfide Synthesis, Metabolism, and Measurement: Is Modulation of Hydrogen Sulfide a Novel Therapeutic for Cancer?** 2019, **31:**1-38.

6.	Guo F-F, Yu T-C, Hong J, Fang J-Y: **Emerging Roles of Hydrogen Sulfide in Inflammatory and Neoplastic Colonic Diseases.** 2016, **7**.

7.	Zhao K, Ju Y, Li S, Altaany Z, Wang R, Yang G: **S-sulfhydration of MEK1 leads to PARP-1 activation and DNA damage repair.** *EMBO reports* 2014, **15:**792-800.

8.	Zhang D, Du J, Tang C, Huang Y, Jin H: **H2S-Induced Sulfhydration: Biological Function and Detection Methodology.** 2017, **8**.

9.	Lee HK, Hsu AK, Sajdak J, Qin J, Pavlidis P: **Coexpression analysis of human genes across many microarray data sets.** *Genome research* 2004, **14:**1085-1094.

10.	Schönbrodt FD, Perugini M: **At what sample size do correlations stabilize?** *Journal of Research in Personality* 2013, **47:**609-612.

11.	Wang L, Jhee K-H, Hua X, DiBello PM, Jacobsen DW, Kruger WD: **Modulation of Cystathionine &#x3b2;-Synthase Level Regulates Total Serum Homocysteine in Mice.** 2004, **94:**1318-1324.

12.	Watanabe M, Osada J, Aratani Y, Kluckman K, Reddick R, Malinow MR, Maeda N: **Mice deficient in cystathionine beta-synthase: animal models for mild and severe homocyst(e)inemia.** *Proc Natl Acad Sci U S A* 1995, **92:**1585-1589.

13.	Grimm SA, Shimbo T, Takaku M, Thomas JW, Auerbach S, Bennett BD, Bucher JR, Burkholder AB, Day F, Du Y, et al: **DNA methylation in mice is influenced by genetics as well as sex and life experience.** *Nature Communications* 2019, **10:**305.

14.	Lee HO, Wang L, Kuo YM, Gupta S, Slifker MJ, Li YS, Andrews AJ, Kruger WD: **Lack of global epigenetic methylation defects in CBS deficient mice.** *J Inherit Metab Dis* 2017, **40:**113-120.

15.	Kruger WD: **Cystathionine β-synthase deficiency: Of mice and men.** *Molecular genetics and metabolism* 2017, **121:**199-205.

16.	Niwa T, Tsukamoto T, Toyoda T, Mori A, Tanaka H, Maekita T, Ichinose M, Tatematsu M, Ushijima T: **Inflammatory processes triggered by Helicobacter pylori infection cause aberrant DNA methylation in gastric epithelial cells.** *Cancer Res* 2010, **70:**1430-1440.

17.	Etchegaray J-P, Mostoslavsky R: **Interplay between Metabolism and Epigenetics: A Nuclear Adaptation to Environmental Changes.** *Molecular Cell* 2016, **62:**695-711.

18.	Yang R, Qu C, Zhou Y, Konkel JE, Shi S, Liu Y, Chen C, Liu S, Liu D, Chen Y, et al: **Hydrogen Sulfide Promotes Tet1- and Tet2-Mediated Foxp3 Demethylation to Drive Regulatory T Cell Differentiation and Maintain Immune Homeostasis.** *Immunity* 2015, **43:**251-263.

19.	Liu Y, Mayo MW, Nagji AS, Smith PW, Ramsey CS, Li D, Jones DR: **Phosphorylation of RelA/p65 promotes DNMT-1 recruitment to chromatin and represses transcription of the tumor metastasis suppressor gene BRMS1.** *Oncogene* 2012, **31:**1143-1154.

20.	O'Hagan HM, Wang W, Sen S, Destefano Shields C, Lee SS, Zhang YW, Clements EG, Cai Y, Van Neste L, Easwaran H, et al: **Oxidative damage targets complexes containing DNA methyltransferases, SIRT1, and polycomb members to promoter CpG Islands.** *Cancer cell* 2011, **20:**606-619.

21.	García-Giménez JL, Pallardó FV: **Maintenance of glutathione levels and its importance in epigenetic regulation.** *Frontiers in pharmacology* 2014, **5:**88-88.

22.	Ulanovskaya OA, Zuhl AM, Cravatt BF: **NNMT promotes epigenetic remodeling in cancer by creating a metabolic methylation sink.** *Nature chemical biology* 2013, **9:**300-306.

23.	Di Simone N, Maggiano N, Caliandro D, Riccardi P, Evangelista A, Carducci B, Caruso A: **Homocysteine Induces Trophoblast Cell Death with Apoptotic Features1.** *Biology of Reproduction* 2003, **69:**1129-1134.

24.	Lin N, Qin S, Luo S, Cui S, Huang G, Zhang X: **Homocysteine induces cytotoxicity and proliferation inhibition in neural stem cells via DNA methylation in vitro.** 2014, **281:**2088-2096.

25.	Akoglu B, Milovic V, Caspary WF, Faust D: **Hyperproliferation of homocysteine-treated colon cancer cells is reversed by folate and 5-methyltetrahydrofolate.** *Eur J Nutr* 2004, **43:**93-99.

26.	Karpinski P, Pesz K, Sasiadek MM: **Pan-cancer analysis reveals presence of pronounced DNA methylation drift in CpG island methylator phenotype clusters.** *Epigenomics* 2017, **9:**1341-1352.

27.	Koch A, Jeschke J, Van Criekinge W, van Engeland M, De Meyer T: **MEXPRESS update 2019.** *Nucleic Acids Research* 2019, **47:**W561-W565.

28.	Takano N, Sarfraz Y, Gilkes DM, Chaturvedi P, Xiang L, Suematsu M, Zagzag D, Semenza GL: **Decreased expression of cystathionine β-synthase promotes glioma tumorigenesis.** *Molecular cancer research : MCR* 2014, **12:**1398-1406.

29.	Fack F, Tardito S, Hochart G, Oudin A, Zheng L, Fritah S, Golebiewska A, Nazarov PV, Bernard A, Hau A-C, et al: **Altered metabolic landscape in IDH-mutant gliomas affects phospholipid, energy, and oxidative stress pathways.** *EMBO molecular medicine* 2017, **9:**1681-1695.

30.	Cancer Genome Atlas Research N: **Comprehensive molecular characterization of gastric adenocarcinoma.** *Nature* 2014, **513:**202-209.

31.	Moarii M, Reyal F, Vert J-P: **Integrative DNA methylation and gene expression analysis to assess the universality of the CpG island methylator phenotype.** *Human Genomics* 2015, **9:**26.

32.	Qu Y, Dang S, Hou P: **Gene methylation in gastric cancer.** *Clin Chim Acta* 2013, **424:**53-65.

33.	Merry CR, Forrest ME, Sabers JN, Beard L, Gao X-H, Hatzoglou M, Jackson MW, Wang Z, Markowitz SD, Khalil AM: **DNMT1-associated long non-coding RNAs regulate global gene expression and DNA methylation in colon cancer.** *Human Molecular Genetics* 2015, **24:**6240-6253.

34.	Cao S, Zhu X, Zhang C, Qian H, Schuttler H-B, Gong J, Xu Y: **Competition between DNA Methylation, Nucleotide Synthesis, and Antioxidation in Cancer versus Normal Tissues.** 2017, **77:**4185-4195.

35.	Tang B, Mustafa A, Gupta S, Melnyk S, James SJ, Kruger WD: **Methionine-deficient diet induces post-transcriptional downregulation of cystathionine β-synthase.** *Nutrition* 2010, **26:**1170-1175.

36.	Machado JC, Oliveira C, Carvalho R, Soares P, Berx G, Caldas C, Seruca R, Carneiro F, Sobrinho-Simöes M: **E-cadherin gene (CDH1) promoter methylation as the second hit in sporadic diffuse gastric carcinoma.** *Oncogene* 2001, **20:**1525-1528.

37.	Leung SY, Yuen ST, Chung LP, Chu KM, Chan AS, Ho JC: **hMLH1 promoter methylation and lack of hMLH1 expression in sporadic gastric carcinomas with high-frequency microsatellite instability.** *Cancer Res* 1999, **59:**159-164.

38.	Yu D, Cao T, Han Y-D, Huang F-S: **Relationships between MGMT promoter methylation and gastric cancer: a meta-analysis.** *OncoTargets and therapy* 2016, **9:**6049-6057.

39.	Stampone E, Caldarelli I, Zullo A, Bencivenga D, Mancini FP, Della Ragione F, Borriello A: **Genetic and Epigenetic Control of CDKN1C Expression: Importance in Cell Commitment and Differentiation, Tissue Homeostasis and Human Diseases.** *International journal of molecular sciences* 2018, **19:**1055.

40.	Miller BF, Sanchez-Vega F, Elnitski L: **The Emergence of Pan-Cancer CIMP and Its Elusive Interpretation.** *Biomolecules* 2016, **6**.

41.	Matsusaka K, Kaneda A, Nagae G, Ushiku T, Kikuchi Y, Hino R, Uozaki H, Seto Y, Takada K, Aburatani H, Fukayama M: **Classification of Epstein-Barr virus-positive gastric cancers by definition of DNA methylation epigenotypes.** *Cancer Res* 2011, **71:**7187-7197.

42.	Zouridis H, Deng N, Ivanova T, Zhu Y, Wong B, Huang D, Wu YH, Wu Y, Tan IB, Liem N, et al: **Methylation subtypes and large-scale epigenetic alterations in gastric cancer.** *Sci Transl Med* 2012, **4:**156ra140.

43.	Nadel J, Athanasiadou R, Lemetre C, Wijetunga NA, Ó Broin P, Sato H, Zhang Z, Jeddeloh J, Montagna C, Golden A, et al: **RNA:DNA hybrids in the human genome have distinctive nucleotide characteristics, chromatin composition, and transcriptional relationships.** *Epigenetics & Chromatin* 2015, **8:**46.

44.	Abakir A, Giles TC, Cristini A, Foster JM, Dai N, Starczak M, Rubio-Roldan A, Li M, Eleftheriou M, Crutchley J, et al: **N6-methyladenosine regulates the stability of RNA:DNA hybrids in human cells.** *Nature Genetics* 2020, **52:**48-55.

45.	Shima H, Matsumoto M, Ishigami Y, Ebina M, Muto A, Sato Y, Kumagai S, Ochiai K, Suzuki T, Igarashi K: **S-Adenosylmethionine Synthesis Is Regulated by Selective N6-Adenosine Methylation and mRNA Degradation Involving METTL16 and YTHDC1.** *Cell Reports* 2017, **21:**3354-3363.

46.	Cedar H, Bergman Y: **Linking DNA methylation and histone modification: patterns and paradigms.** *Nat Rev Genet* 2009, **10:**295-304.

47.	Greer EL, Shi Y: **Histone methylation: a dynamic mark in health, disease and inheritance.** *Nature Reviews Genetics* 2012, **13:**343-357.

48.	Wang C, Oshima M, Sato D, Matsui H, Kubota S, Aoyama K, Nakajima-Takagi Y, Koide S, Matsubayashi J, Mochizuki-Kashio M, et al: **Ezh2 loss propagates hypermethylation at T cell differentiation–regulating genes to promote leukemic transformation.** *The Journal of Clinical Investigation* 2018, **128:**3872-3886.

49.	Dekkers KF, van Iterson M, Slieker RC, Moed MH, Bonder MJ, van Galen M, Mei H, Zhernakova DV, van den Berg LH, Deelen J, et al: **Blood lipids influence DNA methylation in circulating cells.** *Genome Biology* 2016, **17:**138.

50.	Ye C, Sutter BM, Wang Y, Kuang Z, Tu BP: **A Metabolic Function for Phospholipid and Histone Methylation.** *Molecular cell* 2017, **66:**180-193.e188.

51.	Uličná L, Paprčková D, Fáberová V, Hozák P: **Phospholipids and inositol phosphates linked to the epigenome.** *Histochemistry and Cell Biology* 2018, **150:**245-253.

52.	Kuykendall JR, Cox R: **1-Methylnicotinamide stimulates cell growth, causes DNA hypermethylation and inhibits induced differentiation of Friend erythroleukemia cells.** In.; 1992

53.	Huang KK, Ramnarayanan K, Zhu F, Srivastava S, Xu C, Tan ALK, Lee M, Tay S, Das K, Xing M, et al: **Genomic and Epigenomic Profiling of High-Risk Intestinal Metaplasia Reveals Molecular Determinants of Progression to Gastric Cancer.** *Cancer Cell* 2018, **33:**137-150 e135.

54.	Nephew KP, Balch C, Skalnik DG: **Methyl group acceptance assay for the determination of global DNA methylation levels.** *Methods Mol Biol* 2009, **507:**35-41.

55.	Gao F, Ji G, Gao Z, Han X, Ye M, Yuan Z, Luo H, Huang X, Natarajan K, Wang J, et al: **Direct ChIP-bisulfite sequencing reveals a role of H3K27me3 mediating aberrant hypermethylation of promoter CpG islands in cancer cells.** *Genomics* 2014, **103:**204-210.

**Second round of review**

**Reviewer 2**

I went through the responses and happy to say that the authors have addressed the concerns. As mentioned earlier, in the revision, I wanted to highlight some changes to language and interpretations as CIMP is a well-known phenomenon which is intriguing, and most likely occurs via multiple mechanisms. One has to be very careful generalizing this across cancers as there are no universal mechanisms; in fact even no universal mechanisms for DNA methylation alteration in a given tissue type. I want to congratulate the authors for presenting a novel concept.

From me, please consider this as an acceptance of the manuscript in its current format.

**Reviewer 3**

I have read the responses to the reviewer questions again and I am good with the responses.

Two of my recent questions are worth asking based on the presentation of the revised manuscript:

1.	Expression doesn’t seem to change that much in figure 2 B between CIMP+ and nonCIMP, the RSEM is ~8 vs 9? While significant, what is the FD?

2.	Figure 3A, of the 6 cancers displaying significant downregulation of CBS in CIMP+ vs non-CIMP tumors, the normals for HNSC and THYM have less or equal CBS expression than CIMP+ and less methylation. This would suggest that methylation isn’t downregulating expression.

**Authors Response**

**Point-by-point responses to the reviewers’ comments:**

Reviewer 1:

*As mentioned earlier, in the revision, I wanted to highlight some changes to language and interpretations as CIMP is a well-known phenomenon which is intriguing, and most likely occurs via multiple mechanisms. One has to be very careful generalizing this across cancers as there are no universal mechanisms; in fact even no universal mechanisms for DNA methylation alteration in a given tissue type. I want to congratulate the authors for presenting a novel concept.*

*From me, please consider this as an acceptance of the manuscript in its current format.*

Response: We thank the Reviewer for accepting the manuscript in its current format.

Reviewer 2:

*I have read the responses to the reviewer questions again and I am good with the responses.*

Response: We thank the Reviewer for the positive feedback and the comprehensive re-review of our manuscript.

*Two of my recent questions are worth asking based on the presentation of the revised manuscript:*

1. *Expression doesn’t seem to change that much in figure 2 B between CIMP+ and nonCIMP, the RSEM is ~8 vs 9? While significant, what is the FD?*

Response: Thank you for this comment. The TCGA expression values in Figure 2B are log transformed RSEM values from MEXPRESS. The linear fold change between gastric CIMP and EBV CIMP vs non-CIMP are 0.33 and 0.36 respectively, reflecting a clear downregulation of *CBS* in CIMP groups.

2. *Figure 3A, of the 6 cancers displaying significant downregulation of CBS in CIMP+ vs non-CIMP tumors, the normals for HNSC and THYM have less or equal CBS expression than CIMP+ and less methylation. This would suggest that methylation isn’t downregulating expression.*

Response: Thank you for this comment. For HNSC*,* we speculate that the basal expression of *CBS* in normal tissue may be confounded by certain factors. For example, the HNSC samples in the TCGA study were represented by a mixed set of tumours originating from the oral cavity (62%), oropharynx (12%) and laryngeal (26%) sites with tumour adjacent “normal” being >2cm from tumour site [6]. Most patients were male and heavy smokers. In addition to these factors, it is possible that a proportion of histologically normal HNSC tissues may not be “normal” at the molecular level [7], and may have characteristics of pre-malignant lesions [8], as in the case of the gastric intestinal metaplasia [4] and in other cancer settings [9]. A detailed analysis involving larger and appropriate sample types in HNSC may help clarify the expression status of *CBS*. For THYM, only two normal samples were available making interpretation very limited. Moreover, we do not observe *CBS* promoter hypermethylation in the THYM CIMP group.
